# Thymic expression of IL-4 and IL-15 after systemic inflammatory or infectious Th1 disease processes induce the acquisition of "innate" characteristics during CD8^+^ T cell development

**DOI:** 10.1371/journal.ppat.1007456

**Published:** 2019-01-04

**Authors:** Natalia S. Baez, Fabio Cerbán, Constanza Savid-Frontera, Deborah L. Hodge, Jimena Tosello, Eva Acosta-Rodriguez, Laura Almada, Adriana Gruppi, Maria Estefania Viano, Howard A. Young, Maria Cecilia Rodriguez-Galan

**Affiliations:** 1 Inmunología. CIBICI-CONICET, Facultad de Ciencias Químicas, Universidad Nacional de Córdoba, Córdoba, Argentina; 2 Cancer and Inflammation Program, Center for Cancer Research, National Cancer Institute, Frederick, MD, United States of America; National Institute of Health, UNITED STATES

## Abstract

Innate CD8^+^ T cells express a memory-like phenotype and demonstrate a strong cytotoxic capacity that is critical during the early phase of the host response to certain bacterial and viral infections. These cells arise in the thymus and depend on IL-4 and IL-15 for their development. Even though innate CD8^+^ T cells exist in the thymus of WT mice in low numbers, they are highly enriched in KO mice that lack certain kinases, leading to an increase in IL-4 production by thymic NKT cells. Our work describes that in C57BL/6 WT mice undergoing a Th1 biased infectious disease, the thymus experiences an enrichment of single positive CD8 (SP8) thymocytes that share all the established phenotypical and functional characteristics of innate CD8^+^ T cells. Moreover, through *in vivo* experiments, we demonstrate a significant increase in survival and a lower parasitemia in mice adoptively transferred with SP8 thymocytes from OT I—*T*. *cruzi*-infected mice, demonstrating that innate CD8^+^ thymocytes are able to protect against a lethal *T*. *cruzi* infection in an Ag-independent manner. Interestingly, we obtained similar results when using thymocytes from systemic IL-12 + IL-18-treated mice. This data indicates that cytokines triggered during the acute stage of a Th1 infectious process induce thymic production of IL-4 along with IL-15 expression resulting in an adequate niche for development of innate CD8^+^ T cells as early as the double positive (DP) stage. Our data demonstrate that the thymus can sense systemic inflammatory situations and alter its conventional CD8 developmental pathway when a rapid innate immune response is required to control different types of pathogens.

## Author summary

Murine innate CD8^+^ T cells demonstrate strong cytotoxic capacity during the early phase of certain bacterial and viral infections. Such cells have been reported to be present in both mice and humans but many questions remain as to their differentiation and maturation process. Innate CD8^+^ T cells arise in the thymus and depend on IL-4 and IL-15 for their development. A description of the cellular and molecular mechanisms involved during their thymic development has been obtained from KO mice that lack kinases and transcription factors important for TCR signaling. In these mice, SP8 thymocytes with an innate phenotype are highly enriched over the conventional SP8 cells. Our work describes, for the first time, that in WT mice, thymic IL-4 and IL-15 expression triggered by Th1 infectious processes induce an adequate niche for development of innate rather than conventional CD8^+^ T cells. Our data show that the thymus is able to sense a systemic inflammatory response (probably mediated by systemic IL-12 and IL-18 production) and alter its ontogeny when pathogen control is needed.

## Introduction

The thymus is the primary lymphoid organ where T cell development takes place in the host. In physiological conditions, several T cells lineages arise in the organ including conventional αβT cells, γδT cells, regulatory T cells and NKT cells. Most recently more lineages have been added to the list and these include several types of innate T cells[1–3].

The thymic cellular component not only consists of developing cells, but as reported by our group and other laboratories, a small number of mature peripheral B and T cells normally enter the thymus. Furthermore the number of these mature cells increase under inflammatory conditions[4–7]. In this context, our previous work described that during the acute stage of Th1 inflammatory/infectious processes, e.g. *T*. *cruzi* and *C*. *albicans* infections or systemic LPS treatment, a number of peripheral mature T cells with an activated/memory phenotype (CD44^hi^) are able to re-enter the thymus[7]. Moreover, we obtained similar data from mice that systemically express high levels of IL-12 + IL-18, demonstrating that T cells ingress the thymus in a non Ag-specific fashion depending upon a bystander cytokine storm triggered by the inflammatory process rather than to the pathogen itself[7]. However, the number of CD44^hi^ T cells found in the thymus under these Th1 inflammatory conditions is too large to be solely explained by the ingress of peripheral T cells and is more evident in the SP8 subset that is enriched in CD44^hi^ cells. Thus, we speculated that some of the SP8 CD44^hi^ thymocytes might come from internal thymic development as it has been recently reported that SP8 cells with an activated/memory phenotype (CD44^hi^) normally arise in the thymus as an alternative lineage from conventional SP8 thymocytes[8–13]. These cells have been designated as “innate CD8^+^ T cells” and could represent up to 10% of total SP8 thymocytes in both C57BL/6 and BALB/c mice[9, 13, 14]. Over the years, innate CD8^+^ T cells have been further characterized based on their phenotypic and functional properties[15]. During their thymic maturation, innate CD8^+^ T cells up-regulate CD44 and CD122 expression and also acquire high cytotoxic and cytokine production capacities, while conventional memory T cells adopt these characteristic in secondary lymphoid organs (SLO)[16–18]. Other features of innate CD8^+^ T cells include 1: they exert their cytotoxic activity in an Ag-independent manner, 2: they highly depend upon IL-15 for proliferation and 3: they are able to rapidly produce interferon-gamma (IFNγ) when stimulated by IL-12 and IL-18 as response similar to that of NK cells[19–21].

Innate CD8^+^ T cells have been first described in the thymus of mice that lack certain Tec kinases that are important regulators of the TCR signaling cascade that include ITK[12], RLK[11] or the transcription factor KLF2[14]. Currently, they have been described in several other genetically modified mice where the common pathway leads to increased number of invariant NKT cells that express the transcription factor PLZF[22]. In all such models, IL-4 produced in the steady state by invariant NKT cells (or CD4 T cells) is required for SP8 cells to up-regulate the T-box transcription factor eomesodermin (Eomes), that represent one of the featuring markers of this lineage[9, 12, 14, 23].

In our report, we demonstrate a novel “cell developmental pathway” that occurs in the thymus of C57BL/6 WT mice undergoing an acute Th1 systemic infectious/inflammatory process. We provide strong evidence that after infection with 2 different strains of *Trypanosoma cruzi*, the thymus experiences an enrichment of SP8 with an “innate phenotype”. This phenomenon occurs from conversion of DP thymocytes to innate CD8^+^ cells and the generation of newly SP8 thymocytes with innate characteristics. Interestingly this effect can be reproduced after systemic induction of IL-12 and IL-18, cytokines both known to be expressed during the early phase of a Th1 infectious process[24–26] suggesting that this developmental change in the thymus could be driven by Th1 cytokines triggered during an infectious process rather than by the pathogens themselves.

Importantly, a human CD8^+^ T cell subset with similar characteristics to the murine innate CD8^+^ T cells has been recently described[27]. The fact that these cells are also found in cord blood suggests that in humans, innate CD8^+^ T cells might also develop in the thymus[27]. The authors hypothesize that human innate CD8^+^ T cells may play a role in immune defense during the neonatal to early childhood period until an adequate adaptive immune response is established[9, 27].

## Results

**Fig 1:**
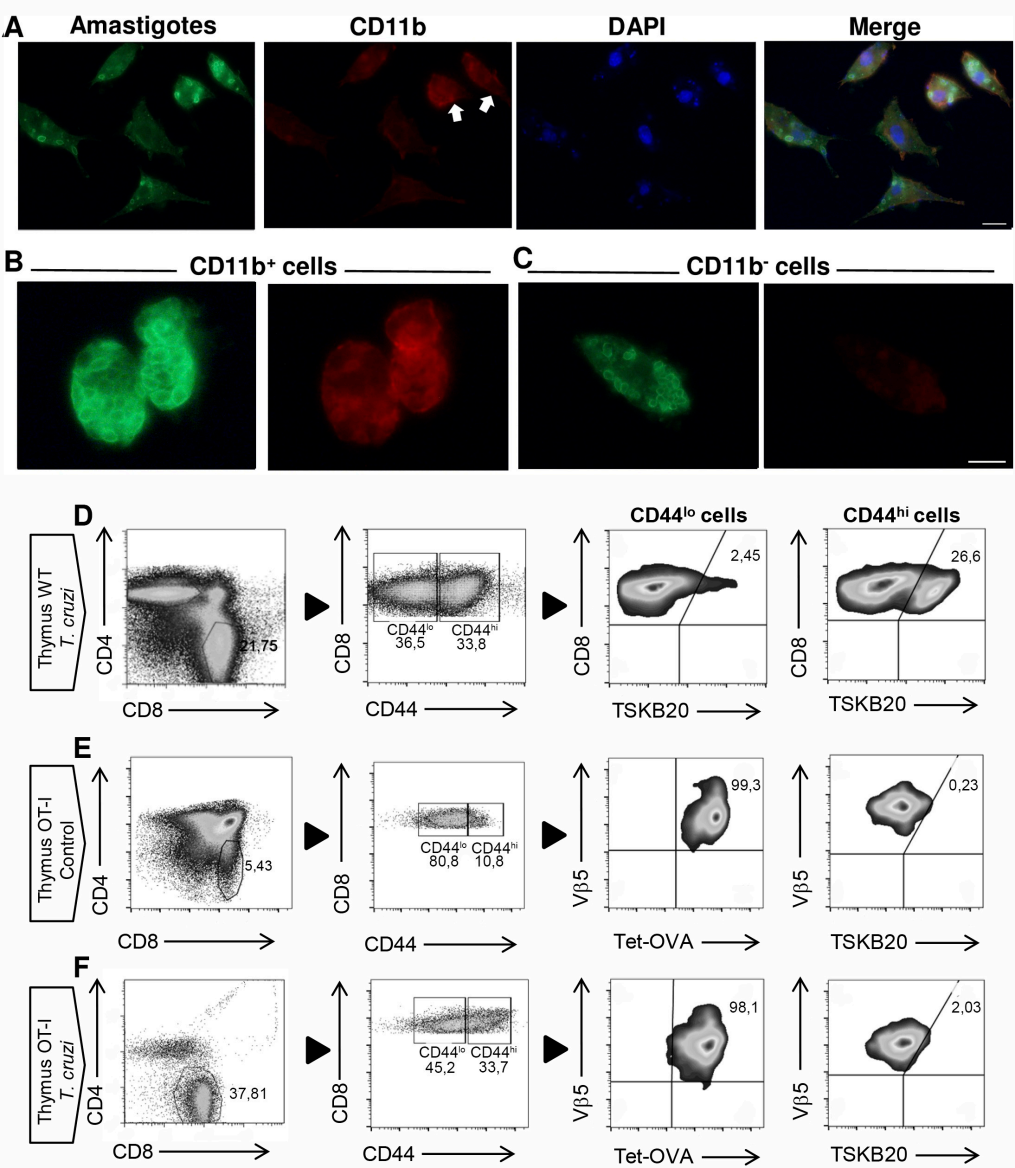
Ag-specific CD8^+^ T cells correlates with the presence of amastigotes in the thymus of *T*. *cruzi*-infected mice. WT mice were infected with *T*. *cruzi* (Tulahuen) and thymic cell suspensions were obtained 14 days post-infection. (A-C) Adherent cells were stained with an antiserum from a chagasic patient and subsequently with an anti-human IgG conjugated with Alexafluor 488. Shown in green are the *T*. *cruzi* parasites, in red (Alexafluor 546) CD11b positive cells and in blue are the nuclei labeled with DAPI. Scale 10 μm. (D) Thymi from *T*. *cruzi*-infected (Tulahuen) WT mice or (E-F) thymi from control or *T*. *cruzi*-infected (Tulahuen) OT-I mice were obtained and surface stained with anti-CD4, anti-CD8, anti-CD44, anti-Vβ5 and an OVA-tetramer or TSKB20-tetramer as described in Material and Methods. Representative dot plots from 2 independent experiments with 5 mice per group are shown.

We have previously demonstrated that migration of mature T cells from SLO to the thymus, that occurs under inflammatory/infectious Th1 processes, is not necessary Ag-driven[7]. However, due to the capacity of *T*. *cruzi* to infect the thymus[28], we speculated that specific T cells might be recirculating to the organ as well. To confirm this hypothesis, we performed immunofluorescence staining, as it has been reported that intracellular amastigotes can be observed inside the infected cells[29]. As hypothesized, Fig 1A shows that *T*. *cruzi* can infect adherent cells from the thymi that are either CD11b^+^ cells (thymic Mϕ, Fig 1B) or CD11b^-^ cells with large and oval-shaped features that resemble thymic fibroblasts (Fig 1C). The presence of the parasite in the thymi of infected mice suggests that Ag-specific T cells might be migrating to the organ. Using a tetramer linked to TSKB20, the most important and immunogenic antigen of the Tulahuen strain of *T*. *cruzi*[30], we evaluated by flow cytometry the presence of specific T cells in the thymi of *T*. *cruzi*-infected WT mice. When we analyzed the SP8 compartment, we observed that the percentage of Ag-specific T cells is higher in the CD44^hi^ cell subset and is very low in the CD44^lo^ cell subset (Fig 1D). This observation is expected as effector/memory T cells express high levels of CD44 after TCR activation[15] (Fig 1D).

TSKB20^+^ cells represent approximately 25% of the total SP8 CD44^hi^ cell in *T*. *cruzi*-infected mice (Fig 1D) and TSKB20 specific T cells are the most abundant T cells during *T*. *cruzi* murine infection with Tulahuen strain[30]. Based on these findings, we investigated what may account for the remaining 75% of SP8 CD44^hi^ cells. We hypothesized 2 different possibilities: 1) Non-Ag specific CD44^hi^ CD8^+^ T cells are arriving to the thymus along with the Ag-specific cells, or 2) A different lineage from conventional T cells could arise in the thymus under these inflammatory Th1 conditions. The latter hypothesis is based on previous reports by several laboratories demonstrating that in mice lacking specific kinases involved in TCR signaling (ITK and RLK KO mice) or the transcription factor KLF2 (KLF2 KO mice), SP8 thymocytes alter their development from the “conventional” to the “innate” lineage[9, 11, 13, 22, 31].

To investigate both options, we first addressed if non-Ag specific cells could account for SP8 CD44^hi^ cells found in the thymus. To avoid a significant alteration in the thymic environment (as we have not identified what signals or cells participate in this phenomenon), we utilized OT-I mice that were not RAG KO. However, we exclusively analyzed cells that were Vβ5^+^/ OVA-tetramer^+^ both in control and in *T*. *cruzi*-infected mice (Fig 1E and 1F, respectively). When we infected OT-I mice with *T*. *cruzi*, we observed an enrichment of CD44^hi^ cells in the SP8 thymic compartment similar to what we detected in WT mice. Moreover, we determined that these cells were OVA specific but TSKB20^neg^ both in uninfected control and *T*. *cruzi*-infected mice (Fig 1E and 1F, respectively).

**Fig 2:**
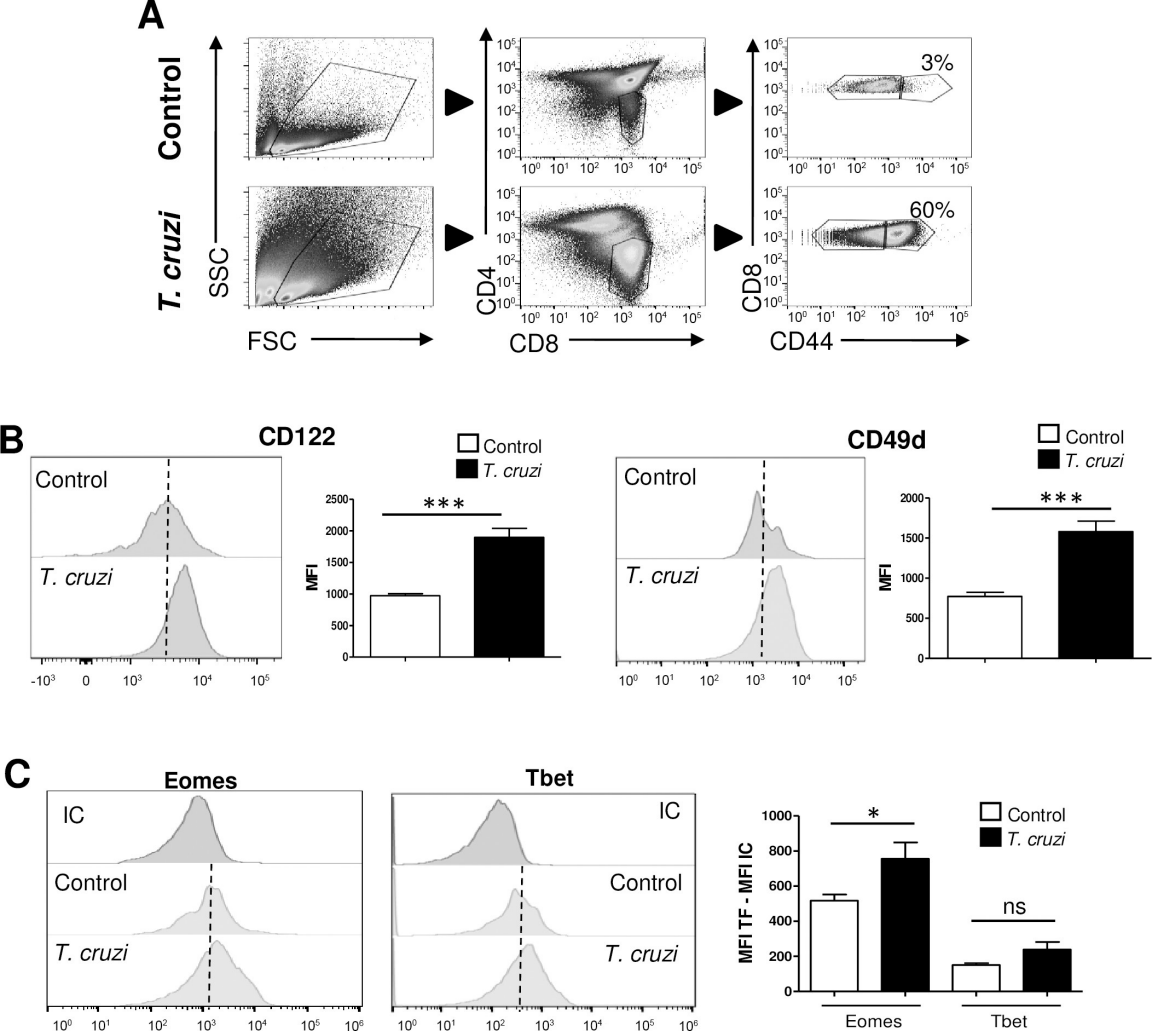
SP8 CD44^hi^ thymocytes from *T*. *cruzi*-infected mice adopt an innate phenotype. (A) Representative dot plots of the gate strategy for SP8 CD44^hi^ thymocytes analysis are shown. The expression of (B) CD122 and CD49d along with (C) Eomes and Tbet was evaluated by flow cytometry in SP8 CD44^hi^ cells from control and *T*. *cruzi*-infected (Tulahuen) WT mice. In (C) transcription factor (TF) expression was expressed as the difference of the mean fluorescence intensity (MFI) of Eomes or Tbet vs the MFI of the corresponding isotype control (IC) in the SP8 CD44^hi^ cells. Student’s unpaired *t* test was used for statistical analysis. Data is representative of 3 repetitions of the same experiment with 3–5 animals per group. Bar graph data are shown as the mean ± SEM. Control versus *T*. *cruzi*-infected mice, *p<0.05, ***p<0.001, NS: non-significant.

Currently, there are no reports demonstrating that a change in the SP8 lineage commitment could occur in WT mice after an infection. In this context, we asked if SP8 CD44^hi^ cells found in the thymus of *T*. *cruzi-*infected mice share characteristics of the innate CD8^+^ T lineage. To test this hypothesis, we performed a flow cytometry phenotypic analysis based on the consensus markers that are known to be expressed by these cells[10, 15, 32, 33]. A main characteristic of the innate CD8^+^ T cells is the expression of CD44. In the case of conventional T cells this marker is acquired by activated or memory T cells in SLO; but in contrast, innate CD8^+^ T cells up-regulate CD44 during their thymic maturation without Ag exposure[9, 13]. Upon gating on SP8 CD44^hi^ cells in the thymi of WT uninfected control or *T*. *cruzi*-infected mice (Fig 2A), we evaluated the CD122 and CD49d surface expression markers. According to what has been previously reported for murine innate CD8^+^ T cells[15], we observed that the bulk population of SP8 CD44^hi^ cells significantly up-regulated the expression of both molecules in *T*. *cruzi*-infected mice as compared to the equivalent population in control mice (Fig 2B). In addition to these two phenotypic markers, murine innate CD8^+^ T cells express high levels of the transcription factor Eomes and exhibit no alteration in Tbet levels. This is contrary to Ag-specific memory cells that up-regulate both factors upon TCR engagement[15, 22]. In our model, we observed that SP8 CD44^hi^ thymocytes in *T*. *cruzi-*infected mice significantly increased Eomes but not Tbet expression compared to the same subset in control mice (Fig 2C). Thus, our results demonstrate that an enrichment of cells with characteristics of innate CD8^+^ T cells occurs in the thymic SP8 compartment after *T*. *cruzi* infection.

As the thymi of WT (B6) mice could also contain Ag-specific cells that share most of the markers of innate CD8^+^ T cells, we infected OT-I mice with *T*. *cruzi* and gated on the Vβ5^+^ cells (we have previously shown in Fig 1 that they all are OVA specific, TSKB20^neg^ cells). Interestingly, we observed that SP8 CD44^hi^ thymocytes express all the features of innate CD8^+^ T cells suggesting that the innate characteristics are acquired in an Ag-independent process (S1A Fig). Moreover, when we used control and *T*. *cruzi*-infected WT along with control and *T*. *cruzi*-infected OT-I mice to compare the percentage of thymic SP8 CD44^hi^ and SP8 CD44^lo^ cells, we observed a similar pattern both in the total cell number and percentage of cells. Additionally, and as expected, the absolute cell numbers were lower in OT-I mice when compared to WT mice (S1B Fig).

A fast screening of different Th1 infectious setting demonstrate an enrichment of SP8 CD44^hi^ Eomes^hi^ cells in the thymi of *C*. *albicans*-infected mice (S2A Fig) and also a large number of SP8 thymocytes with innate features after infection of mice with a different strain of *T*. *cruzi* (strain Y, S2B Fig). Based on these data, we hypothesized that appearance of thymic innate CD8^+^ T cells could be triggered by systemic levels of the cytokines IL-12 and IL-18. We based this hypothesis on knowledge that these infections induce a strong Th1 inflammatory process, resulting in elevated IL-12 and IL-18 production during the acute stage[24–26]. Furthermore, innate CD8^+^ T cells are known to constitutively express the receptor for these cytokines, and respond with high IFNγ production, as previously reported[21, 34]. To test this hypothesis, we induced IL-12+IL-18 systemic expression by cDNA hydrodynamic shear[7, 35, 36] and observed an enrichment of thymic SP8 CD44^hi^ thymocytes with all the characteristics of innate CD8^+^ T cells (S2C Fig).

**Fig 3:**
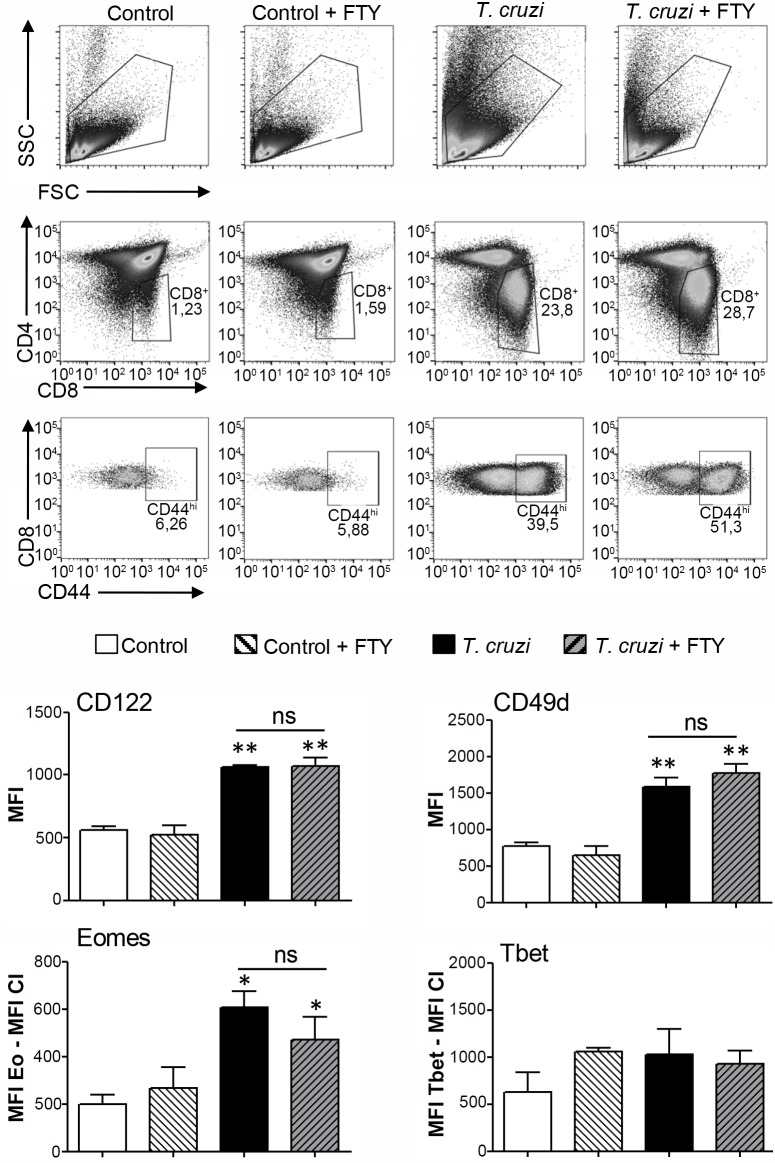
Innate CD8^+^ cells in the thymus of *T*. *cruzi*-infected mice are not derived from SLO. Control or *T*. *cruzi*-infected (Tulahuen) WT mice were treated with FTY720. Individual mice from both groups received 3 25μg injections of FTY720 in 200ul of saline solution on days 8, 10 and 13 post-infection. Control mice received saline only injections on the indicated days. Thymocytes from control or *T*. *cruzi*-infected mice, with and without FTY720 treatment, were obtained 14 days post-infection. The expression of CD122 and CD49d was evaluated by flow cytometry in SP8 CD44^hi^ cells. Eomes and Tbet expression was measured by intranuclear staining using Flow cytometry analysis in the SP8 CD44^hi^ cells and expressed as described in Fig 2. One-way ANOVA was used for statistical analysis. Data are shown as the mean ± SEM. Control versus *T*. *cruzi*-infected mice, *p<0.05, **p<0.01. FTY = FTY720.

Next, to determine the origin of these cells, we performed *in vivo* experiments on *T*. *cruzi*-infected B6 mice treated with and without the immunosuppressant drug fingolimod (FTY720) (Fig 3). FTY720 arrests recirculation of T cells by blocking their exportation from SLO. This is due to internalization and degradation of S1P receptors[37]. Fig 3 shows similar percentages of CD44^hi^ SP8 cells in FTY720 treated and untreated mice that share all innate CD8^+^ T cell markers. This data suggests that most of these cells are of thymic origin.

**Fig 4:**
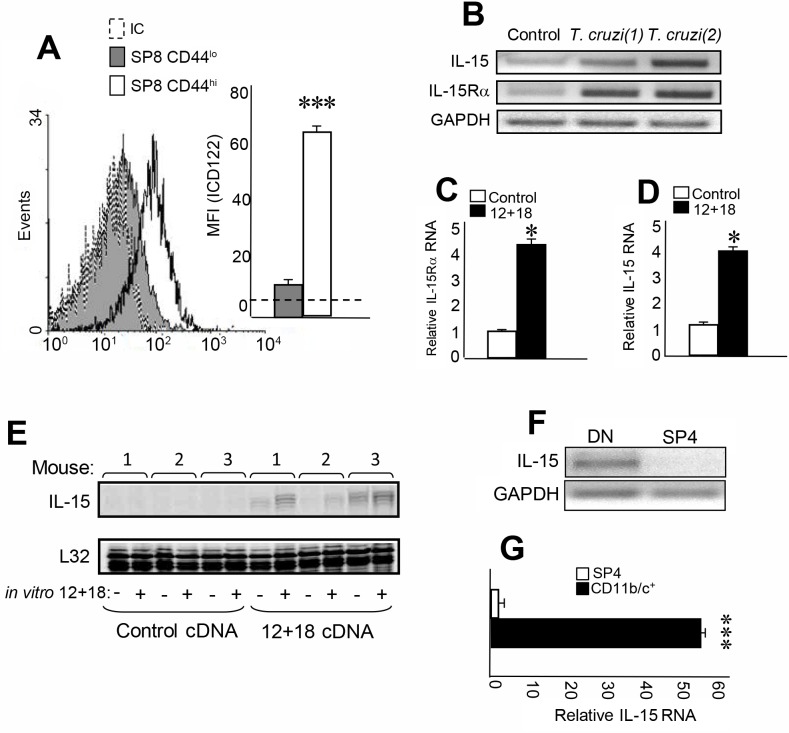
IL-15 is produced in the thymus and its receptors are expressed by SP8 CD44^hi^ thymocytes. Thymocytes from *T*. *cruzi*-infected (Tulahuen) WT mice were obtained 14 days after infection. The expression of (A) IL-2/IL-15 β chain (CD122) in SP8 CD44^hi^ and CD44^lo^ thymocytes was evaluated by Flow cytometry. Histograms are representative from 3 independent experiments with 3–5 mice each. Results are shown as mean ± SEM. The statistical test applied was Student’s unpaired *t* test. SP8 CD44^hi^ versus SP8 CD44^lo^ cells, ***p<0.*001*. (B) IL-15 and IL-15Rα chain RNA expression was evaluated by RT PCR in total thymus from control or *T*. *cruzi*-infected mice. Figure shows one representative control mouse and 2 *T*. *cruzi*-infected mice (1) and (2). (C) IL-15Rα chain and (D) IL-15 RNA expression was evaluated by real time PCR in total thymus from control cDNA or IL-12+IL-18 cDNA-treated mice. Results are shown as mean ± SEM. The statistical test applied was Student’s unpaired *t* test. Control versus IL-12+IL-18 treatment, *p<0.*05*. (E) IL-15 expression was evaluated by RPA in total thymus from control cDNA or IL-12+IL-18 cDNA-treated mice after *in vitro* stimulation (or not) with rIL-12 (10μg/ml) + rIL-18 (50 μg/ml). (F) IL-15 RNA expression was evaluated by RT PCR in the sorted DN or SP4 thymocytes or by (G) Real-time PCR in the sorted CD11b^+^/CD11c^+^ or SP4 cell subset obtained from thymi of *T*. *cruzi*-infected mice. Results are shown as mean ± SEM. The statistical test applied was a Student’s unpaired *t* test, ***p<0.*001*.

As previously mentioned, IL-4 is the key cytokine responsible for Eomes induction during thymic differentiation of innate CD8^+^ T cells[12, 14, 23]. However, another cytokine involved in proliferation and survival of this lineage is IL-15[15, 22]. In fact, it has been reported that innate CD8^+^ T cells expressed high levels of CD122, the IL2/IL-15 β chain receptor along with IL-4Rα[8–13, 16]. Based on this data, we speculated that SP8 CD44^hi^ cells (CD122^hi^ CD49d^hi^ Eomes^hi^) found in *T*. *cruzi*-infected and IL-12 + IL-18 treatment models should express both IL2/IL-15 and IL-4 cytokine receptors. First, we analyzed IL-15, both cytokine and receptor expression. Fig 4A shows that CD122 is almost exclusively expressed by SP8 CD44^hi^ cells compared to the SP8 CD44^lo^ counterpart. We determined that the high affinity α chain of the IL-15 receptor is also expressed in the thymi of *T*. *cruzi*-infected mice (Fig 4B). Interestingly, thymi from IL-12+IL-18-treated mice also express IL-15Rα RNA (Fig 4C). A relevant finding is that IL-15 RNA is expressed in the thymi only in *T*. *cruzi*-infected (Fig 4B) and IL-12+IL-18-treated mice (Fig 4D) but not in control mice. Moreover, IL-15 can be further induced after rIL-12+rIL-18 *in vitro* stimulation of thymi of IL-12+IL-18 cDNA-treated mice but not in control mice (Fig 4E). Next, we evaluated the source of IL-15 in the thymus and found out that IL-15 is expressed in the double negative (DN) thymic compartment (Fig 4F). Moreover, further investigation determined that IL-15 RNA is highly expressed by thymic myeloid CD11b^+^/CD11c^+^ cells, a subset of DN cells (Fig 4G).

**Fig 5:**
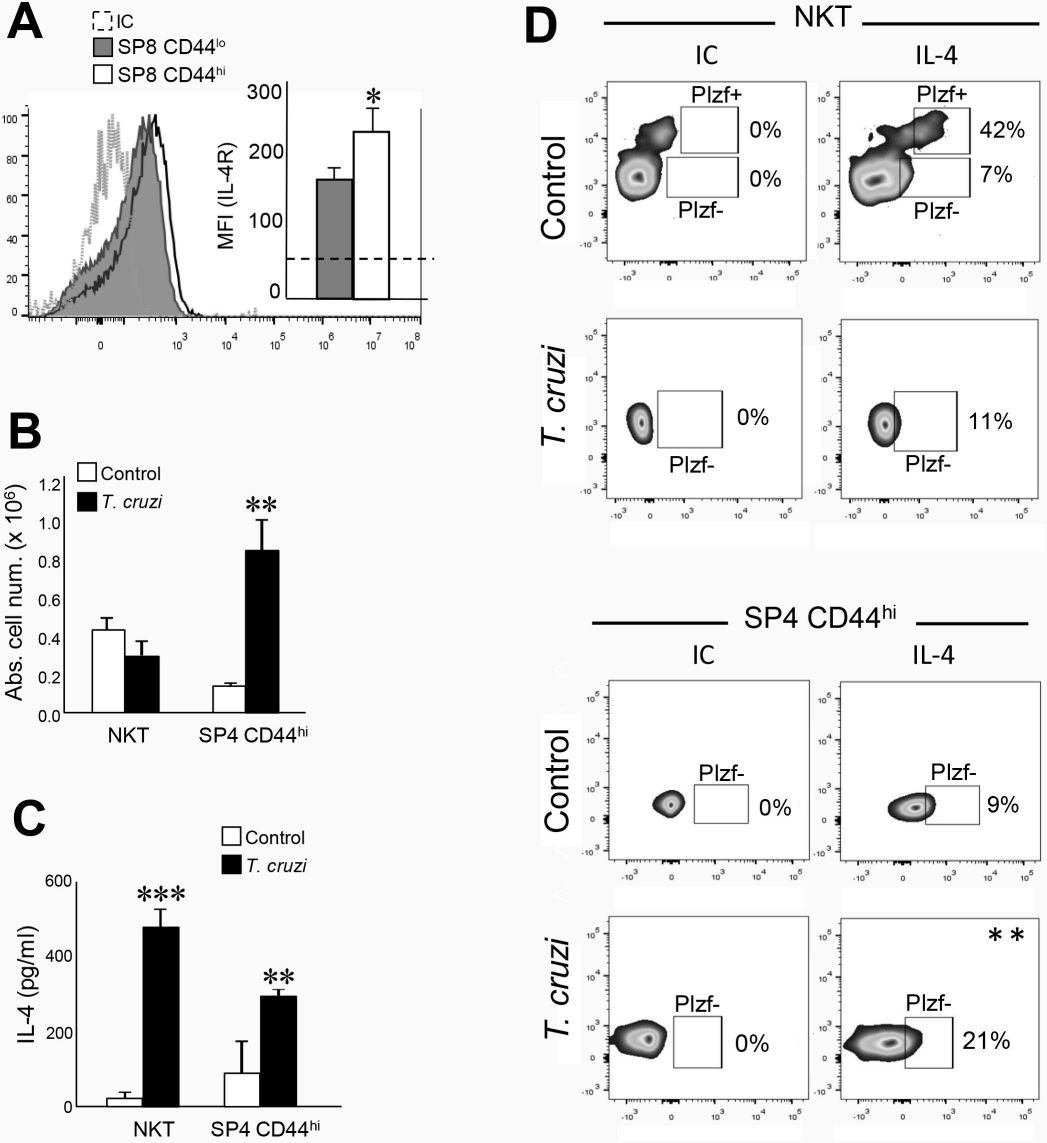
IL-4 is produced in the thymus by several cell subsets and its receptors are expressed by SP8 CD44^hi^ thymocytes. Thymocytes from *T*. *cruzi*-infected (Tulahuen) WT mice were obtained 14 days after infection. The expression of (A) IL-4R in SP8 CD44^hi^ and CD44^lo^ thymocytes was evaluated by Flow cytometry. Histograms are representative from 3 independent experiments with 3–5 mice each. Results are shown as mean ± SEM. The statistical test applied was Student’s unpaired *t* test. SP8 CD44^hi^ versus SP8 CD44^lo^ cells, *p<0.*05*. (B) The absolute cell numbers of thymic NKT and SP4 CD44^hi^ were calculated in control and *T*. *cruzi*-infected mice. Control versus *T*. *cruzi*-infected mice **p<0.*01*. (C) NKT and SP4 CD44^hi^ thymocytes were isolated by cell sorting from thymi of control or *T*. *cruzi*-infected mice (see gate strategy in S3 Fig used to sort cells). Cells were cultured for 5h in complete medium in the presence of PMA (50ng/ml) and Ionomycin (1μg/ml). Production of IL-4 was evaluated in the supernatants by ELISA. IL-4 concentration ± SEM shown is the result of 2 independent experiments. The statistical test applied was a one-way ANOVA. Control versus *T*. *cruzi*-infected mice, **p<0.*01*, ***p<0.*001*. (D) The intracellular expression of IL-4 was evaluated in thymic NKT and SP4 CD44^hi^ thymocytes (see gate strategy in S3 Fig) by Flow cytometry. Dot plots are representative from 3 independent experiments with 2–3 mice each. Results are shown as percentage of IL-4^+^ cells. The statistical test applied was Student’s unpaired *t* test. **p<0.*01*.

In the case of IL-4, we first determined that both SP8 CD44^lo^ and SP8 CD44^hi^ thymocytes from *T*. *cruzi*-infected mice express IL4Rα chain but at levels significantly higher in SP8 CD44^hi^ cells (Fig 5A). When we analyzed the source of IL-4 in the thymus, we focus on NKT cell since it has been reported by several laboratories as the main source of thymic IL-4[22]. However, we did not want to miss other potential IL-4-producing cells like SP CD4 (SP4) CD44^hi^ cells since it has been reported that innate CD8^+^ T cells can developed in the presence of PLZF^+^ CD4^+^ thymocytes[23]. In the case of SP4 CD44^hi^ cells, we have previously reported that they are present in the thymi of *T*. *cruzi*-infected mice[7] and their absolute number are higher than in control mice (Fig 5B).

Then we decided to sort NKT and SP4 CD44^hi^ cells from thymi of control and *T*. *cruzi*-infected mice and determine the functional capacity to produce IL-4 after PMA/Ionomycin *in vitro* stimulation (see gate strategy in S3 Fig). Interestingly, data from Fig 5C demonstrated that thymic NKT and SP4 CD44^hi^ cells from *T*. *cruzi*-infected mice are much higher IL-4 producers than the same population from control mice. To determine if PLZF is involved in IL-4 production in these cells, we performed intracellular IL-4 and PLZF staining. Unfortunately, we could not stimulate the cells with PMA/Ionomycin due to TCR downregulation in NKT cells and the loss of CD1d tetramer detection after activation as previously reported[38]; however, we could confirm that in the thymi of control mice, the most important source of IL-4 was NKT PLZF^+^ cells as reported by several laboratories[22]. Surprisingly, in *T*. *cruzi*-infected mice, NKT PLZF^+^ cells cannot be detected (Fig 5D), although they were able to produce much larger amounts of IL-4 than NKT cells from control mice as shown in Fig 5C. Also, in *T*. *cruzi*-infected mice we detected a larger percentage of IL-4^+^ SP4 CD44^hi^ that are also negative for PLZF (Fig 5D). These results demonstrated that induction of the two relevant cytokines involved in innate CD8^+^ T cell development/survival/proliferation occurred locally in the thymi of mice undergoing a systemic inflammatory/infectious Th1 process. Moreover, after the systemic infectious process, thymic IL-4 was produced by multiple sources that are different from the ones in control mice.

**Fig 6:**
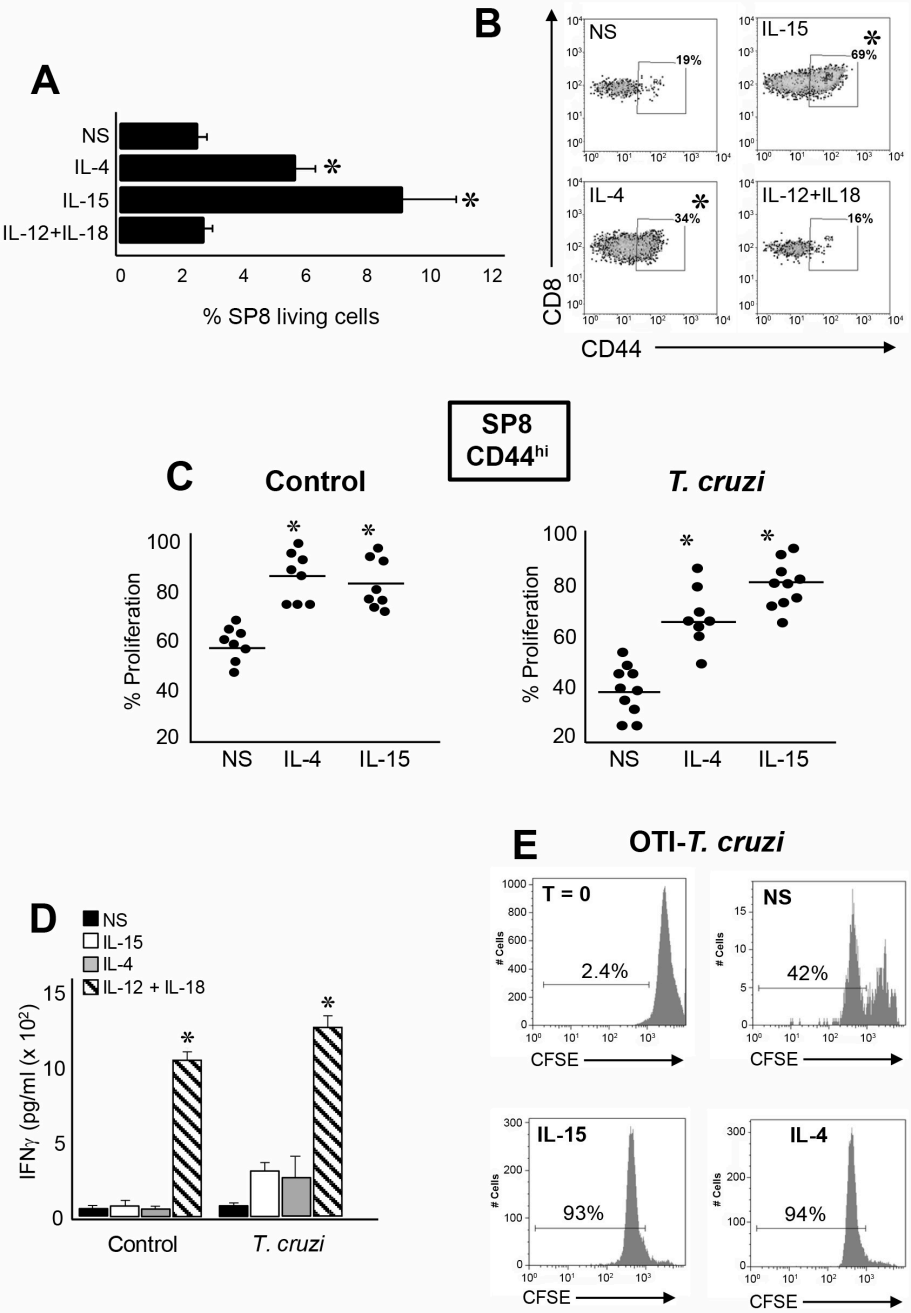
Preferential expansion/selection of innate CD8^+^ thymocytes in the thymus of *T*. *cruzi*-infected mice. Thymocytes from *T*. *cruzi*-infected (Tulahuen) WT mice were cultured with 150 ng/ml of rIL-15, 20 ng/ml of rIL-4 or 10 ng/ml of rIL-12 plus 50 ng/ml of rIL-18. After 72h of culture cells were harvested and Flow cytometry analysis was performed. The percentage of (A) SP8 cells in the living gate of total thymocytes or (B) SP8 CD44^hi^ thymocytes were calculated. In (A) data was expressed as mean ± SEM of 2 independent experiments with 4–5 mice per group, Histograms are representative of one out of 2 independent experiments. NS versus IL-4 or IL-15, *p<0.*05*. (C) Cells from thymi of control or *T*. *cruzi*-infected (Tulahuen) WT mice were stained with 4μM CFSE dye and percent proliferation in the SP8 CD44^hi^ subset was calculated by the CSFE dilution compared to the expression at T = 0 (before the cultures) and analyzed by flow cytometry. (D) IFNγ production was analyzed by ELISA in the culture supernatant from each condition. Results are shown as mean ± SEM, 12+18 versus NS, IL-4 or IL-15, *p<0.*05*. (E) Thymocytes from *T*. *cruzi*-infected (Tulahuen) OT-I mice were stained with 4μM CFSE dye and then cultured with 150 ng/ml of rIL-15, or 20 ng/ml of rIL-4. After 72h, the percentage of proliferation was calculated based on the CSFE dilution analyzed by flow cytometry as explained above and compared with T = 0. Histograms are representative of one OT-I *T*. *cruzi*-infected mouse from two independent experiments with 4–5 mice/group. The statistical test applied was a One-way ANOVA.

We next evaluated the biological effects of IL-4 and IL-15 in innate SP8 thymocytes. When we cultured thymocytes from *T*. *cruzi*-infected mice, we observed an overall survival of SP8 cells only when stimulated with recombinant IL-4 (rIL-4) or rIL-15 but not with rIL-12+rIL-18 (Fig 6A). Moreover, we observed a significant increase in the percentage of SP8 CD44^hi^ cells after rIL-4 and rIL15 stimulation (Fig 6B). Even though SP8 CD44^hi^ cells proliferate under non-stimulated (NS) conditions, the proliferative rate significantly increased in the presence of rIL-4 or rIL-15 in both control and *T*. *cruzi*-infected mice (Fig 6C). We next determined whether IL-4 and IL-15 are able to induce IFNγ production in this lineage, and we observed only a moderate increase in IFNγ in the bulk population of cells from *T*. *cruzi*-infected mice but not in control mice Fig 6D). As an additional experimental control, we stimulated the bulk population of the same thymocytes with IL-12+IL-18 and observed high levels of IFNγ largely due to the fact that they contain NKT, CD8^+^ and CD4^+^ CD44^hi^ cell types known to be high producers of this cytokine (Fig 6D). When we performed a similar experiment using thymocytes from OT-I *T*. *cruzi*-infected mice, we determined that IL-4 and IL-15 are able to induced robust proliferation equal to the polyclonal population of thymocytes (Fig 6E).

**Fig 7:**
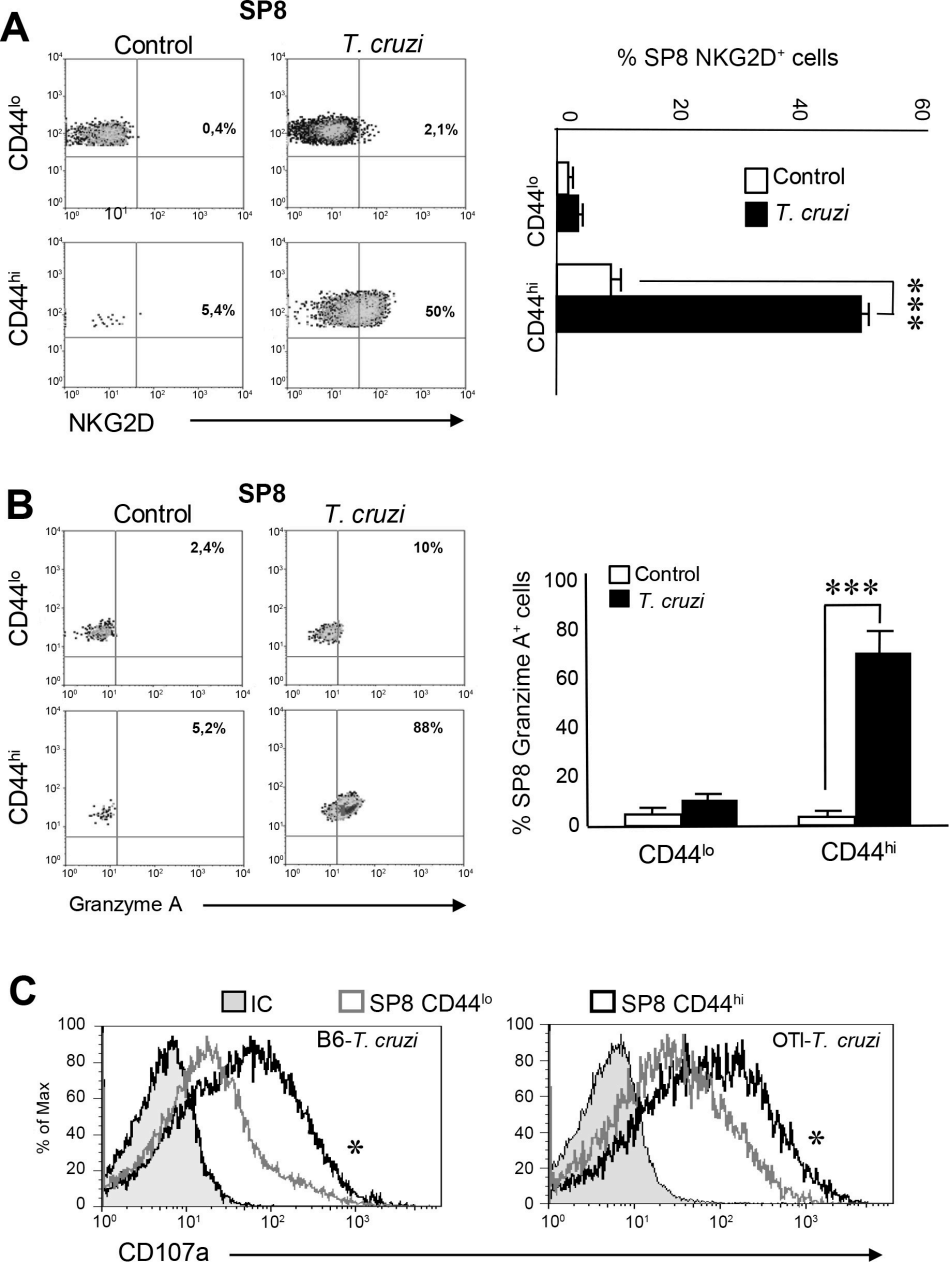
SP8 CD44^hi^ thymocytes from *T*. *cruzi*-infected mice adopt cytotoxic features. (A) The percentage of SP8 NKG2D^+^ cells or (B) SP8 Granzyme A^+^ cells were analyzed by Flow cytometry in CD44^hi^ and CD44^lo^ thymocytes isolated from control or *T*. *cruzi*-infected (Tulahuen) WT mice. Dot plots are representative of one mouse per group from three independent experiments with 4–6 mice per group. The statistical test applied was a One-way ANOVA. Control versus *T*. *cruzi*-infected mice, ***p<0.*001*. (C) Thymi from WT *T*. *cruzi*-infected (Tulahuen) or OT-I *T*. *cruzi*-infected (Tulahuen) mice (at 14 dpi) were isolated and thymocytes were cultured in the presence of PMA/ionomycin, Brefeldin A and an anti-CD107a Ab for 5h in complete media. Flow cytometry analysis was performed to evaluate the expression of CD107a in the subpopulations SP8 CD44^hi^ (black line) and SP8 CD44^lo^ (gray line), *p<0.*05*. The statistical test applied was a One-way ANOVA. Histograms are representative of two independent experiments with 3–5 mice/group.

It has been demonstrated that innate CD8^+^ T cells have a potent TCR-independent cytotoxic activity that involves granzymes and perforins release and NKG2D receptor-driven killing activity [19, 39–41]. Moreover, these cells play an important role during the early control of certain bacterial and viral infections[20, 21, 39, 42, 43]. In this context, we evaluated the expression of NKG2D in the SP8 compartment of control and *T*. *cruzi*-infected mice. Interestingly, NKG2D is highly up-regulated in SP8 CD44^hi^ (CD122^hi^ CD49d^hi^ Eomes^hi^) thymocytes only after *T*. *cruzi* infection but its expression was detected in the equivalent population in control mice (Fig 7A). Similar results were observed with granzyme A as it was highly expressed only in SP8 CD44^hi^ (CD122^hi^ CD49d^hi^ Eomes^hi^) thymic cells of *T*. *cruzi*-infected mice (Fig 7B). Moreover, SP8 CD44^hi^ (CD122^hi^ CD49d^hi^ Eomes^hi^) cells from *T*. *cruzi*-infected mice demonstrated a high CD107a expression after PMA stimulation that correlates with a higher degranulation capacity than the CD44^lo^ counterpart cells (Fig 7C, left panel). Similar results were observed when we measured CD107a expression on SP8 CD44^hi^ vs SP8 CD44^lo^ cells in OT-I *T*. *cruzi*-infected mice (Fig 7C, right panel). These results led us to speculate that thymic SP8 innate cells may exert a protective role during *T*. *cruzi* infection in a similar manner as has been reported for peripheral innate T cells in other murine infection models[20, 21, 39, 42, 43]. Production of IFNγ has been reported to be involved during protective immunity against *Trypanosoma cruzi* infection[44, 45]. Interestingly, IFNγ is a key cytokine that is highly produced by innate CD8^+^ cells[20–22]. In our *T*. *cruzi* infection model, we found that thymic SP8 CD44^hi^ cells from *T*. *cruzi*-infected OT-I mice produce much higher amounts of IFNγ compared to SP8 CD44^hi^ cells from the control group or SP8 CD44^lo^ cells from both groups of mice(S4 Fig). Moreover, IFNγ^+^ cells correlated with Eomes expression (S4 Fig). This is consistent with the observation that Eomes was first reported to be the critical transcription factor promoting IFNγ expression in innate CD8^+^ T cells[13, 16].

**Fig 8:**
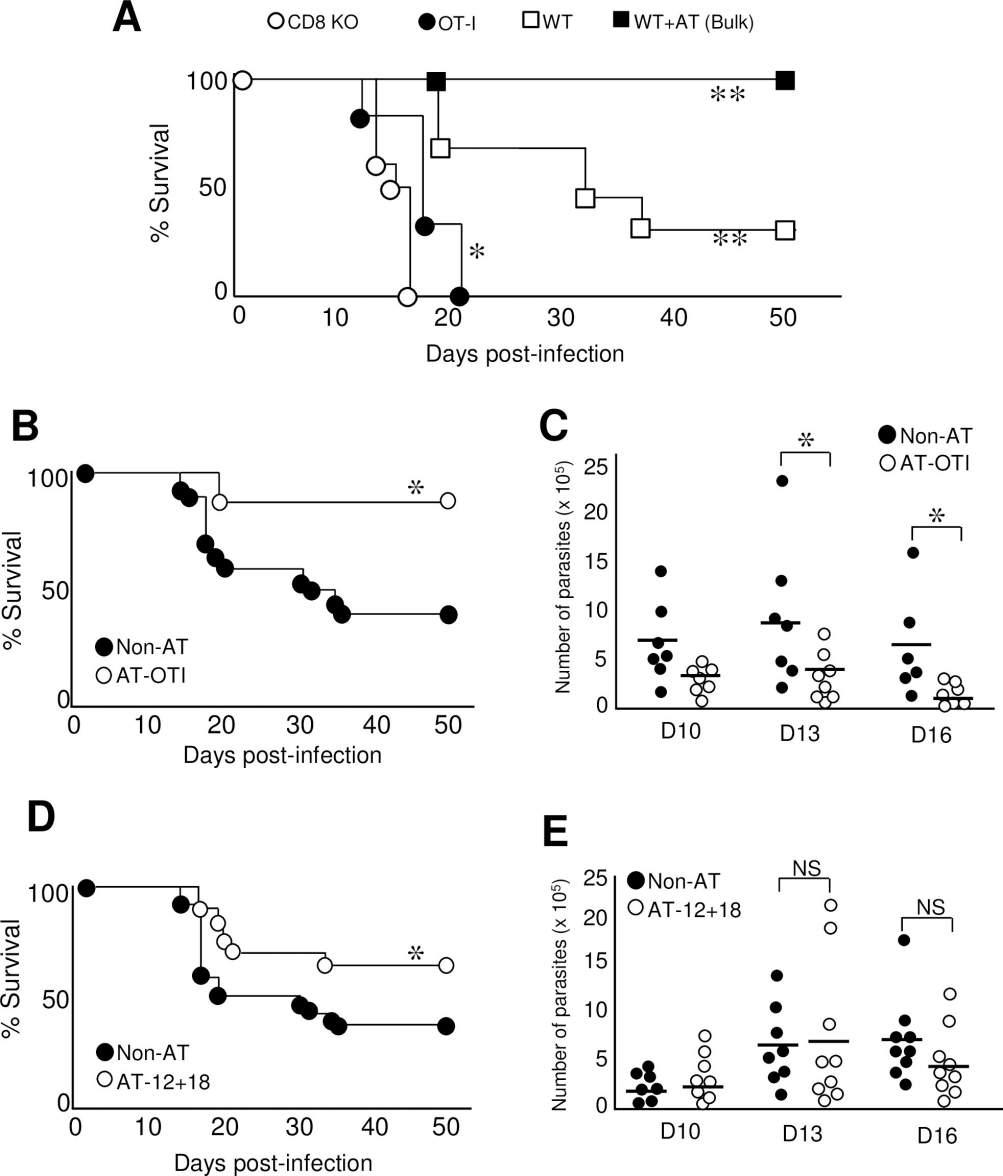
Innate CD8^+^ thymocytes induce protection during *T*. *cruzi* infection in an Ag-independent manner. (A) CD8αKO, OT-I and WT mice were infected with 5000 *T*. *cruzi* parasites (Tulahuen) (recipient mice). In the WT-AT group, a bulk suspension of 10 x 10^6^ thymocytes from *T*. *cruzi-* infected mice was adoptively transferred (AT) to WT B6 mice 24h prior to infection with 5000 *T*. *cruzi* parasites. Survival was compared between groups and monitored at different time points after infection. WT+AT, WT or OT-I vs CD8KO, *p<0.*05*, **p<0.*01* (B) WT mice were not AT (Non-AT) or AT with sorted 5–6 x 10^6^ SP8 thymocytes from OT-I *T*. *cruzi*-infected (Tulahuen) mice (AT-OTI) 24h prior to the infection with 5000 *T*. *cruzi* parasites and monitored for survival daily post infection (C) Parasitemia (number of parasites per ml of blood) of non-AT or AT-OTI was evaluated on days 10, 13 and 16 post infection. (D) Non-AT mice or mice AT with a bulk suspension of 10 x10^6^ thymocytes from IL-12+IL-18 cDNA-treated mice (AT-12+18) were monitored for survival in the days post infection with 5000 *T*. *cruzi* parasites. (E) Parasitemia of non-AT or AT-12+18 was evaluated on days 10, 13 and 16 post infection. Data represent two replicates of the same experiment with 6–8 animals per group. The number of parasites was compared with a One-way ANOVA test and survival data were analyzed with the Wilcoxon-Gehan-Brelow test. Non-AT versus OTI-AT or AT-12+18, *p<0.*05*. NS: not significant. WT = conventional C57BL/6 mice.

Ag-specific CD8^+^ T are known to be crucially protective during the immune response against *T*. *cruzi*[46]. To investigate if Ag-independent CD8^+^ T cells are also able to exert protection in this infection model, we performed survival experiments using WT, CD8 KO and OT-I mice challenged with 5000 tripomastigotes. Survival was evaluated over 50 days post-infection utilizing a protocol described elsewhere[47]. As expected, CD8 KO mice died rapidly after infection compared to WT mice that carry Ag-specific and non-specific CD8^+^ T cells (Fig 8A). OT-I mice also died faster than WT mice but surprisingly survived better than CD8 KO mice, indicating that the CD8^+^ T cells present that are non-specific for this parasite could still induce some protection (Fig 8A). This result encouraged us to investigate if thymic innate CD8^+^ cells could also induce protection in this model. First we adoptively transferred (AT) a bulk population of thymocytes obtained from *T*. *cruzi*-infected mice (donors) to *T*. *cruzi*-infected mice (recipients) and observed 100% survival of recipient mice (Fig 8A). Since the bulk population of thymocytes obtained from *T*. *cruzi*-infected mice have both specific and non-specific CD8^+^ T cells (as demonstrated above, Fig 1) along with other cell types, we carried out survival experiments by transferring only SP8 thymocytes (>90% cells with innate CD8^+^ phenotype) from *T*. *cruzi*-infected OT-I mice to *T*. *cruzi*-infected mice. We observed a significant increase in survival along with a significant diminution of parasitemia in AT-OTI compared to non-AT recipient mice (Fig 8B and 8C, respectively). To evaluate if protection can be performed by innate CD8^+^ T cells from thymi of pathogen free mice, we adoptively transferred thymocytes obtained from IL-12+IL-18-treated mice and observed a significant increase in survival compared to non-AT mice (Fig 8D), although no changes in the parasitemia was observed (Fig 8E).

Thus far, our data have demonstrated that under systemic Th1 conditions, the thymus experiences changes in its cellular composition in a manner that accounts for an enrichment of innate SP8 cells over the conventional cell types. These cells share all the phenotypic and functional characteristics that identify innate CD8^+^ T cells and we have further demonstrated that adoptive transfer of these innate CD8^+^ thymocytes exerts protection during *T*. *cruzi* infection in an Ag-independent manner.

**Fig 9:**
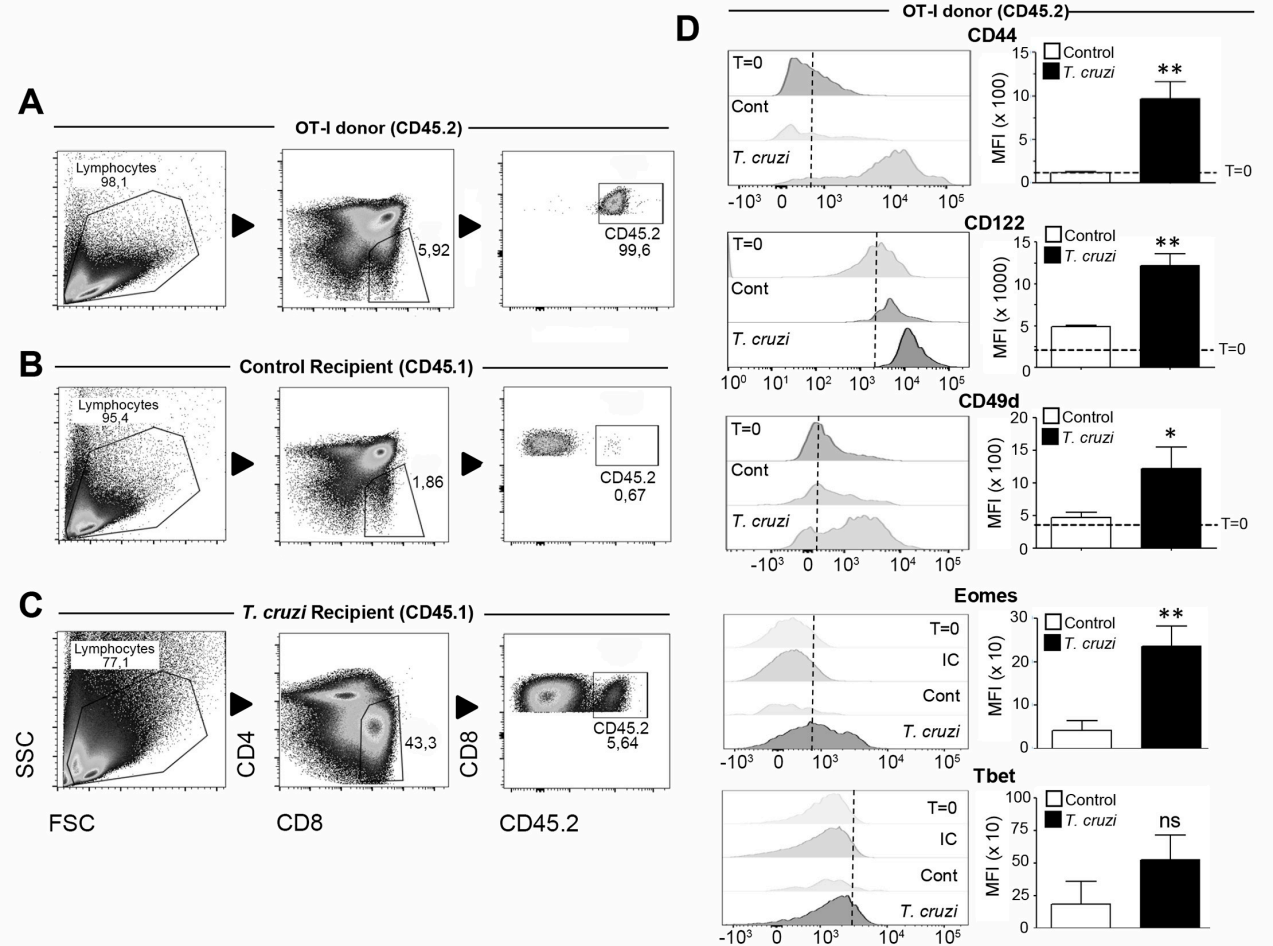
Innate CD8^+^ cells appearance in the thymus is a SP8 lineage decision. CD45.1^+^ Control or *T*. *cruzi-*infected (Tulahuen) WT mice at day 12 post-infection (recipient mice) were anaesthetized and i.t. injected with 10 x 10^6^ CD45.2^+^ thymocytes from the same control OT-I mouse (donor). After 48h, mice were sacrificed and the thymi were harvested. Dot plot shows the gate strategy for analysis of: (A) CD45.2^+^ SP8 OT-I thymocytes before being injected and after 48 h of being injected in (B) control or (C) *T*. *cruzi-*infected mice. Two days post i.t. injections (D) CD44, CD122, CD49d, Eomes and Tbet expression were analyzed by Flow cytometry in the CD45.2^+^ SP8 gated OT-I thymocytes. Changes in Eomes and Tbet levels were expressed as the difference between the MFI of Eomes or Tbet and the MFI from the corresponding Isotype control (IC). Histograms are representative of 2 independent experiments with 4–6 mice/group. The statistical test applied was a Student’s unpaired *t* test, Control vs T-cruzi *p<0.*05* and **p<0.*01*.

**Fig 10:**
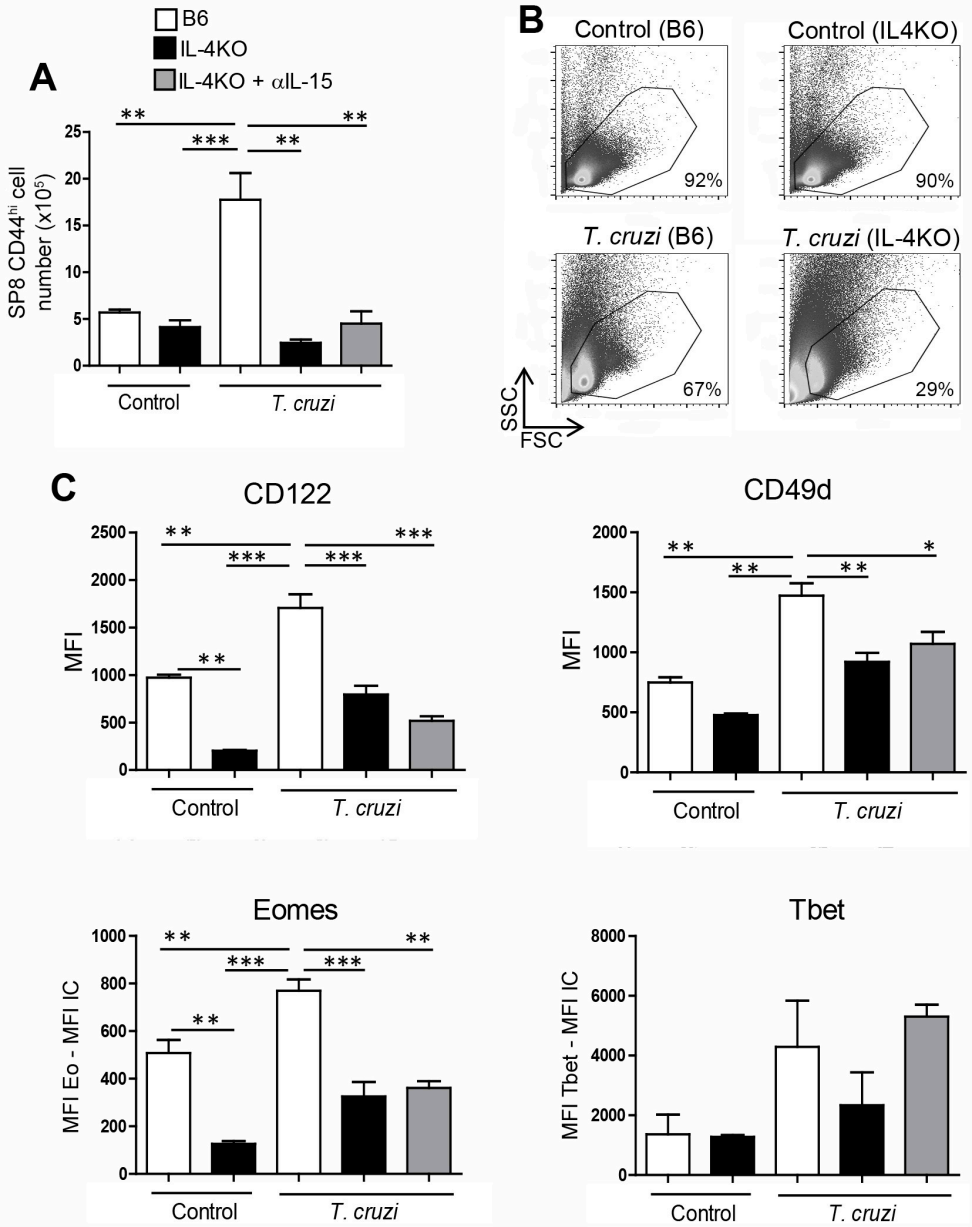
IL-4 expression is important to generate innate CD8+ thymocytes after *T*. *cruzi* infection. WT and IL-4KO mice were infected with *T*. *cruzi* (Tulahuen) and sacrificed 14 days post-infection (dpi). IL-4KO *T*. *cruzi*-infected mice were separated in 2 different groups, one with no treatment and the other was treated with 2 i.p. injections of anti-IL15 antibody (25 μg of IL-15 per injection) at 10 and 12 dpi. Thymocytes were obtained on day 14 dpi and (A) the total number of SP8 CD44^hi^ cells was calculated. (B) Representative density plots of one mouse per group that shows the percentage of total viable cells. (C) The expression of CD122 and CD49d was evaluated by flow cytometry in SP8 CD44^hi^ cells. Eomes or Tbet were measured by intranuclear staining using Flow cytometry analysis expression and were expressed as the difference of the mean fluorescence intensity (MFI) of Eomes or Tbet vs the MFI of the correspondent isotype control (IC) in the SP8 CD44^hi^ subset. Data are shown as the mean ± SEM. The statistical test applied was a One-way ANOVA. *T*. *cruzi* vs the rest of the groups, *p<0.*05*,**p<0.01 and ***p<0.*001*.

A relevant question that remains to be answered in this work is the origin of innate CD8^+^ cells found in the thymi of mice undergoing Th1 infectious/inflammatory processes. As a result of the FTY720 experiment (Fig 3) we hypothesized that these cells should be generated in the thymus. To address this question we utilized three different strategies. Our first approach was to stain the bulk population of thymocytes by performing intrathymic (i.t.) injections with the eFluor 670 (eF670) dye, in control and *T*. *cruzi*-infected mice at day 7 post-infection. We selected this time point as we already determined it to be the latest point when both groups of mice still contained the same proportion and phenotype of both SP8 CD44^lo^ and SP8 CD44^hi^ thymocytes (S5A Fig). Seven days later (day 14), when innate CD8^+^ cells were largely abundant in the thymus of *T- cruzi*-infected mice, we analyzed the thymi of both groups and observed that while SP8 eF670^+^ cells in control mice still maintained the original phenotype, SP8 eF670^+^ cells in *T*. *cruzi*-infected mice had significantly increased CD44 expression (S5B Fig). This data, along with the FTY720 experiments, represented the first indication that most innate SP8 cells found in the thymus may result from an endogenous conversion/expansion rather than migration from SLO. To confirm this hypothesis, we developed a second strategy by performing i.t. injections of CD45.2^+^ thymocytes from a control OT-I mouse (donor) into 2 different thymic environments: a CD45.1^+^ control mouse or a CD45.1^+^ *T*. *cruzi*-infected mouse (both B6 recipients). After 48h, we obtained the thymi and analyzed CD45.2^+^ Vβ5^+^ (OVA specific) cells in both groups of recipient mice. Fig 9A, 9B and 9C show the gate strategy used to analyze only CD45.2 expression of OT-I transferred donor cells. After the i.t. injections, we observed that the SP8 CD45.2^+^ cell numbers recovered in control recipient CD45.1^+^ mice were significantly lower than in *T*. *cruzi*-infected recipient mice (Fig 9B and 9C, respectively). When the phenotype of transferred donor CD45.2 cells was analyzed, we observed that except for Tbet, all innate CD8^+^ T cell markers were up-regulated only when injected into *T*. *cruzi*-infected recipient mice (Fig 9D). This data strongly demonstrated that during a systemic Th1 process like *T*. *cruzi* infection, the conventional SP8 thymic compartment becomes enriched in innate CD8^+^ cells. This led us to hypothesize that IL-4 and IL-15 could be responsible for this effect. Thus, we performed *in vivo* experiments with control or *T*. *cruzi*-infected mice in the absence of both cytokines. Interestingly, before the infection, the absolute number of SP8 CD44^hi^ cells in IL-4KO mice was not changed as compared to B6 mice (Fig 10A). However, after *T*. *cruz*i infection, the outcome was totally different; while SP8 CD44^hi^ cells were significantly increased in B6 mice, the cell number greatly dropped in IL-4KO mice due to an overall decrease in thymic cell viability in these mice (Fig 10B). Furthermore, expression of CD122, CD49d in SP8 CD44^hi^ cells was diminished in IL-4KO *T*. *cruzi*-infected mice contrary to what was observed in B6 mice where these markers increased after infection (Fig 10C). It is worth to mention that while the total cell number in uninfected condition was similar between B6 and IL-4KO mice, both Eomes and CD122 expression was significantly lower in IL-4KO mice (Fig 10C). This is consistent with a previous report demonstrating that IL-4 up-regulates Eomes that, in turn, up-regulates CD122 expression[16]. These *in vivo* experiments confirmed that simultaneous neutralization of both IL-4 and IL-15 did not exacerbate the robust effects already triggered by the lack of IL-4 alone.

The *in vivo* neutralizing experiment provided substantial information especially about the role of IL-4 in innate CD8^+^ thymic development; however, it could not discern whether systemic or local (thymic) IL-4 and IL-15 were ultimately responsible for the generation of these cells in *T*. *cruzi*-infected mice. Another essential question that remains was whether innate CD8^+^ cells arise from pre-existing conventional SP8 cells or from earlier stages in the T cell development. To test this question, we developed an *in vitro* model based on a previous report[48]. We sorted DP cells from B6 (WT) or OT-I control CD45.2^+^ mice and co-cultured them with the bulk population of thymocytes from either CD45.1^+^ control or CD45.1^+^ *T*. *cruzi*-infected mice. We harvested the co-cultures 48h later and focused only on CD45.2^+^ cells (S6A Fig). Flow cytometry analysis demonstrated that SP8 cells but not SP4 cells arose from the DP cultures and, only in the presence of thymocytes from control or *T*. *cruzi*-infected mice but not when cultured alone from both WT or OT-I mice (S6B Fig). Moreover, our data demonstrated that OT-I DP cells either pre (CD69^neg^) or post-selection (CD69^pos^) generated equivalent large numbers of SP8 cells (S6B Fig).

**Fig 11:**
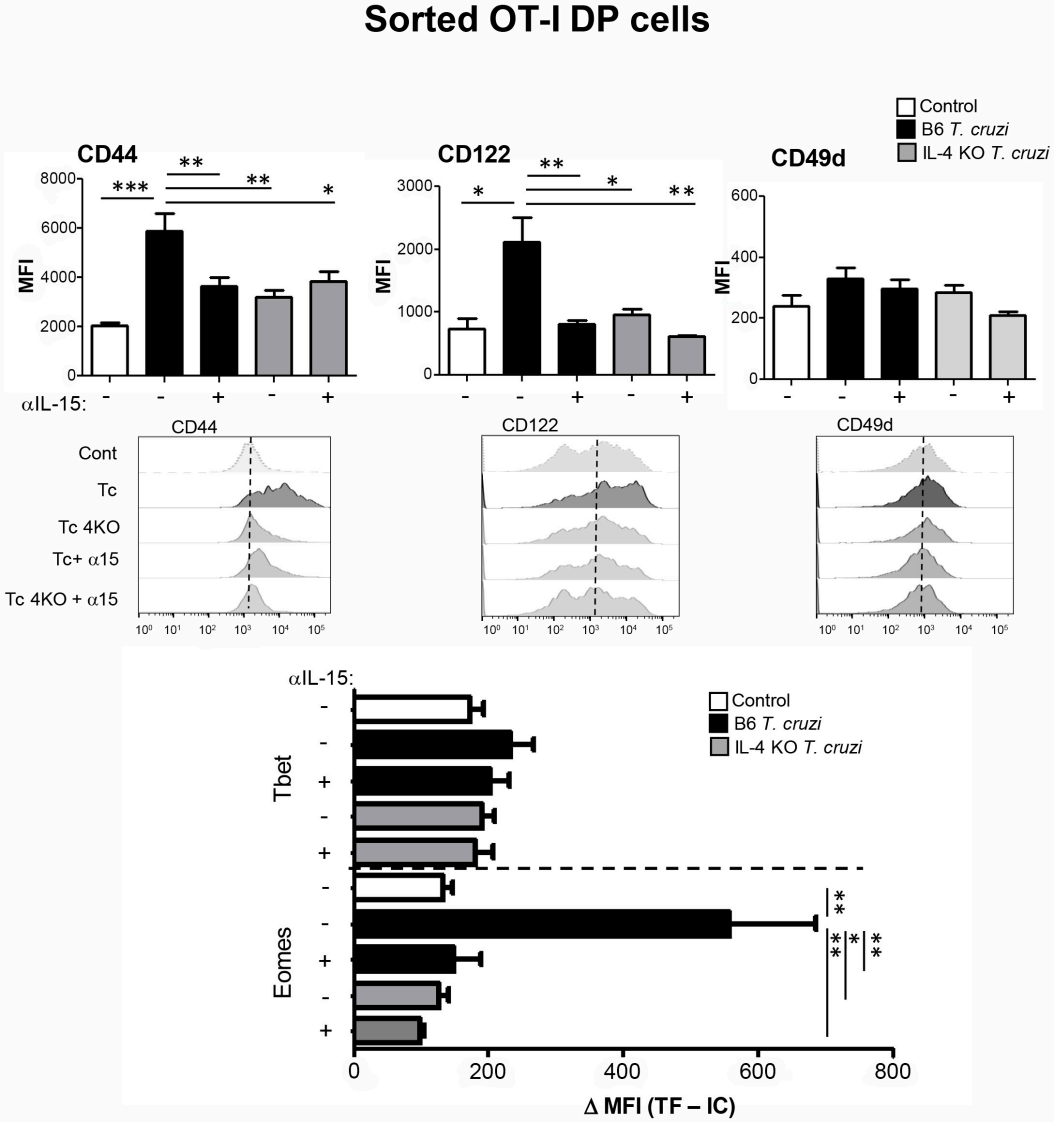
Blockage of IL-4 and IL-15 inhibits the induction of the innate phenotype in DP thymocytes. A bulk population of CD45.2^+^ thymocytes either from WT control, WT *T*. *cruzi-*infected (Tulahuen) or IL-4KO *T*. *cruzi-*infected (Tulahuen) mice were obtained at day 14 post-infection and cultured for 2h at 37°C in the presence of PMA/ionomycin. Cells were washed twice and co-cultured with sorted DP cells from OT-I (Vβ5^+^ OVA-tetramer^+^) control mice at a 1:1 ratio in the presence or absence of a neutralizing anti-IL-15 Ab. After 48h, thymocytes were obtained and CD44, CD122, CD49d, Eomes and Tbet expression were analyzed by Flow cytometry only in DP CD8 OVA-specific OT-I thymocytes. Eomes or Tbet were measured by intranuclear staining using Flow cytometry analysis and were expressed as the difference of the mean fluorescence intensity (MFI) of Eomes or Tbet vs the MFI of the correspondent isotype control (IC). Histograms are representative of two independent experiments with 3–6 mice/experiment. The statistical test applied was a One-way ANOVA. *T*. *cruzi* vs the rest of the groups, *p<0.*05*, **p<0.01 and ***p<0.*001*. Tc = *T*. *cruzi*; Tc+α15 = *T*. *cruzi* + anti-IL-15 neutralizing Ab; Tc4KO = IL-4 KO *T*. *cruzi*; Tc4KO+α15 = IL-4 KO *T*. *cruzi* + anti-IL-15 neutralizing Ab.

When we analyzed the phenotype of sorted DP cells from OT-I mice, we observed that cells that were in contact *in vitro* with thymocytes from *T*. *cruzi*-infected mice were able to adopt innate CD8^+^ features (except for CD49d expression that remained unchanged) (Fig 11). In order to evaluate the role of local IL-4 and IL-15 in the development of thymic innate CD8^+^ T cells, we performed the same co-cultures with WT (B6) and IL-4KO (B6) mice treated with and without anti-IL-15 neutralizing antibody. Data shown in Fig 11 demonstrated that both cytokines are equally important in the acquisition of the innate phenotype. However, the simultaneous blocking of both cytokines did not show any additive effect. Interestingly, when we analyzed SP8 cells generated in the DP cultures, we observed once again, that the cells acquired an innate phenotype (except for CD49d) only when co-cultured with thymocytes from *T*. *cruzi-*infected mice that is inhibited in the absence of IL-4 or IL-15 (S7 Fig). The lack of expression of the commonly associated marker CD49d in the *in vitro* model may indicate that other signals are required for a full innate CD8^+^ phenotype that is acquired in the *in vivo* models but not *in vitro*. Finally, we evaluated sorted SP8 CD44^lo^ thymocytes under the same co-culture conditions and observed only a slightly increase in CD44 and Eomes between control and *T*. *cruzi* co-cultures that did not revert in the absence of IL-4 and IL-15. This suggested that at this more mature stage of development, conversion to the innate phenotype and vice versa was not as flexible as in the DP stage (S8 Fig).

This data demonstrated that a systemic Th1 infection like *T*. *cruzi* was able to trigger thymic production of IL-4 and IL-15, that in turn, facilitated the appearance of SP8 thymocytes with an innate phenotype. This change in thymic development demonstrated greater flexibility when thymocytes were more immature (e.g. the DP stage) and not possible when the thymocytes acquired the more mature SP8 phenotype. Moreover, preliminary data with *C*. *albicans* and systemic IL-12+IL-18 encourages us to investigate whether this phenomenon is relevant to other infectious pathological processes that trigger a strong systemic Th1 cytokine response.

## Discussion

Development of CD8^+^ T cells in the thymus generates a predominant population of conventional naïve cells, along with minor populations of “innate” T cells that resemble memory cells. When analyzing the innate populations that arise in the thymus in a variety of KO mice that have an impaired TCR signaling pathway, several studies have demonstrated the presence of an increased number of IL-4-dependent innate CD8^+^ T cells (as compiled by Lee et al.[9]). These KO mouse models all converge on the fact that a population of thymic cells (PLZF^+^: NKT, γδ T cells or CD4 CD44^hi^ cells) ultimately produces increased levels of IL-4 that drives innate CD8^+^ T cell development[9, 11, 12, 14]. However, the question remains as to whether a similar pathway regulates innate CD8^+^ T cell development in normal mice. Interestingly, inbred strains of mice were shown to vary in their frequency of IL-4-producing invariant NKT (iNKT) cells, with BALB/c mice on top of the spectrum and C57BL/6 mice on the low end. This data that correlates with higher percentages of SP8 CD44^hi^ CD122^hi^ Eomes^hi^ innate cells in the thymus of BALB/c mice compared to C57BL/6 mice under physiological conditions[49]. Moreover, the importance of IL-4-producing iNKT cells in innate CD8^+^ cells development in the thymus is supported by the fact that no innate CD8^+^ T cells are found in BALB/c IL-4R KO and Cd1d KO mice[14].

Surprisingly, at the present time, there are no reports that address patho-physiological conditions that preferentially drive innate CD8^+^ T cell development over conventional CD8^+^ T cells in the thymus. In this context, our work presents strong evidence that an infectious process, like *Trypanosoma cruzi* infection, triggers a systemic Th1 response that leads to an enrichment of SP8 cells phenotypically expressing CD44^hi^, CD122^hi^ CD49d^hi^ Eomes^hi^ Tbet^int/lo^[9, 10, 13, 15, 32, 33, 50] and with functional characteristics (NKG2D^hi^, Granzyme^hi^, CD107a^hi^)[19, 20, 39–42] of innate CD8^+^ T cells.

Interestingly, the acquisition of the innate phenotype occurs in the thymus environment and no from recirculation of mature peripheral T cells as demonstrated by the FYT720 and the co-culture experiments. Moreover, since no significant changes were observed in the number of SP8 CD44^hi^ cells between FYT720-treated and untreated mice, we hypothesize that the large percentage of SP8 CD44^hi^ TKSB20^+^ cells inside the thymus could result from migration of few mature Ag-specific cells from SLO that enter the thymus. However, when inside, they might proliferate and even acquire an innate phenotype due to local IL-4 and IL-15 expression. This data is supported by studies perform and reported by our laboratory indicating that small numbers of mature peripheral T cells are able to enter the thymus in infectious/inflammatory situations[7]. While it has been reported that *T*. *cruzi* infection in mice can alter multiple aspects of thymic biology[28, 51], we demonstrate here that the infectious conditions that trigger the appearance of thymic innate CD8^+^ cells are due to the bystander cytokine storm resulting from the systemic Th1 infectious processes. In support of this hypothesis, we observed similar results in three different experimental settings: 1) mice infected with two different *T*. *cruzi* strains (Tulahuen and Y), 2) in OT-I *T*. *cruzi*- infected mice (pathogen-independent model) and 3) in the absence of infection as in IL-12+IL-18 systemically treated mice.

Some reports describe that interaction of thymocytes with non-conventional MHC-Ib molecules expressed by thymic hematopoietic cells are important for innate CD8^+^ T cell development[23, 52]. Even though we cannot eliminate the possibility that cell-cell interactions are important for innate CD8^+^ T cell development, we demonstrate that innate CD8^+^ thymocytes are almost completely reverted to conventional SP8 thymocytes in the absence of both IL-4 and IL-15. Moreover, it appears that local production of IL-4 and IL-15 expression by different subsets of thymic cells plays a non-redundant role in innate CD8^+^ T cell development. In this context, it is important to emphasize that selection processes that occur in the thymus are not all TCR dependent. In fact, during lineage selection, some maturation events are strictly driven by cytokines but not by the TCR, especially by the γc chain-dependent cytokines (e.g. IL-7 is known to impose CD8 lineage fate)[53]. Thus, it is not unexpected that triggered expression of IL-4 and IL-15 in the thymus by these inflammatory situations could alter the normal/conventional lineage commitment of SP8 thymocytes. This is especially relevant since IL-4 is the extrinsic factor that induces Eomesodemin expression, the key transcription factor associated with innate CD8^+^ T cells[9, 12, 14, 23]. Furthermore, innate CD8^+^ T cells are also dependent on IL-15 signaling for their development and maintenance as innate CD8^+^ T cells, similar to NK cells, are largely absent in IL-15-deficient mice[54]. Moreover, a previous report indicates that one week after *in vivo* administration of an IL-15 blocking antibody, there is a significant reduction in the percentage of innate CD8^+^ T cells in ITK-deficient mice[55].

The *in vitro* experiments also reinforce the finding that the innate phenotype does not occur by homeostatic expansion of resident SP8 CD44^hi^ cells due to available space resulting from the death of DP cells that occurs after the infection. This is demonstrated by our co-culture experiments where we seeded the same numbers of WT or OT-I thymocytes in the plate and obtained similar outcomes as in the *in vivo* intrathymic experiments. Moreover, we determined that less mature thymocytes (e.g. DP cells) are more “flexible” in their ability to adopt the innate CD8^+^ T cell features than the already pre-existing mature SP8 thymocytes. However, from the *in vitro* model we demonstrated that SP8 thymocytes that develop from DP cells tend to easily adopt the innate rather than the conventional SP8 phenotype. Interestingly, our findings are supported by other reports demonstrating that following *T*. *cruzi* infection in BALB/c mice, high levels of IL-4 are produced in the thymus and this finding correlates with the appearance of CD44^hi^ DP cells[28, 56].

Conversion of a naïve CD8^+^ T cells to the innate phenotype may also occur in SLO in the presence of IL-4 as reported by others[13, 18, 57, 58]. Moreover, it has been demonstrated that even conventional αβ memory CD8 T cells are able to exert innate-like functions in response to heterologous challenge (e.g. infections) and are independent of cognate antigen recognition[58]. This cytokine-driven phenomenon occurs when memory CD8 T cells are spatially positioned close to pathogen-activated macrophages and phagocytes in lymph nodes (LNs) and efficiently receive IL-12, IL-15 along with inflammasome-generated IL-18 signals. This cytokine interaction induces a rapid Ag-independent IFNγ expression and effector functions by conventional αβ memory CD8^+^ T cells[58]. These observations not only blur the strict distinction between the innate and adaptive immune compartments but also challenge the established paradigm that innate and adaptive immune responses are undertaken by different type of cells.

Innate CD8^+^ T cells acquire effector function during their maturation process in the thymus rather than by interaction with specific antigens in SLO[13, 32]. It has been postulated that they exert their cytotoxic capacity in a TCR-independent manner by mechanisms that involve strong and rapid production of IFNγ, killing activity through receptors like NKGD2 and degranulation of granzymes and perforins[19, 20, 39–42]. Moreover, it has been reported that peripheral innate CD8^+^ T cells play an important role during the early stages of certain bacterial and viral infections[20, 21, 39, 42, 43]. Our data show that innate CD8^+^ cells that develop in the thymus of *T*. *cruzi*-infected mice not only up-regulate those receptors but also, when adoptively transferred to *T*. *cruzi*-infected mice, exert protection in an Ag-independent manner. This was demonstrated by the survival experiments with adoptive transferred SP8 cells from OT-I *T*. *cruzi*-infected mice or from IL-12+IL-18-treated mice. Our work provides new and unique data about the role of these cells during this parasite infection suggesting that they might effectively operate during the early control of several types of infections, a role previously reported in certain bacterial and viral infections models[20, 21, 39, 42, 43].

Currently, we are performing experiments designed to demonstrate that the innate CD8^+^ T cells that develop under these systemic inflammatory/infectious processes are able to exit the thymus and induce protection in SLO. Preliminary data suggest that, exportation of CD8^+^ T cells with an innate phenotype is observed in these models albeit in lower numbers than in control mice. However, reduction in the exportation of cells from the thymus to SLO is not a concern since it has been extensively reported that this is a common phenomenon during systemic infections[59]. Even though those experiments suggest that innate CD8^+^ T cells might reach SLO under these conditions, at present we are addressing the question as to whether upon cessation of the inflammatory/infectious process, is the thymus able to recover its normal anatomical and cellular components and return to the development of conventional CD8^+^ T cells.

An important aspect to take into account is that CD8^+^ T cells that share a similar phenotype with their innate murine counterpart have been recently described in humans[27]. This report describes a subset of CD8^+^ T cells KIR/NKG2A^+^ CD8^+^ T cells in healthy human adults with increased Eomes expression, prompt IFNγ production in response to innate-like stimulation by IL-12+IL-18, and a potent Ag-independent cytotoxicity[27]. The investigators also identified this cell type in human cord blood, suggesting that development did not depend on cognate antigens and likely arises from the thymus as well[27]. Furthermore, another recent report demonstrates that a higher number of peripheral innate CD8^+^ T cells correlates with a better outcome in certain cancer patients[15, 50].

Overall, our work contributes to the understanding that the thymus is not an isolated and immune privileged organ, but rather has the capacity to sense peripheral stimuli and adapt its developmental program to meet the real time immunological needs. Furthermore, studies to demonstrate that this phenomenon also occurs in humans need to be pursued in order to better understand immune developmental mechanisms and to develop approaches to harness immune responses to fight infections and cancer.

## Materials and methods

### Ethics statement

The experimental protocols were approved by the Institutional Animal Care and Use Committee (IACUC) from Facultad de Ciencias Químicas, Univesidad Nacional de Córdoba, (authorization no. 2016–249). This committee follows the guidelines for animal care in the “Guide to the care and use of experimental animals” (Canadian Council on Animal Care, 1993) and the “Institutional Animal Care and Use Committee Guidebook” (ARENA/OLAW IACUC Guidebook, Nacional Institutes of Health, 2002). Our animal facility also has obtained NIH animal welfare assurance (assurance number A5802-01, OLAW, NIH, USA).

### Mice

Female and male WT C57BL/6 CD45.2^+^, WT C57BL/6 CD45.1^+^, OT-I (RAG-sufficient, B6 background), and CD8KO mice (B6 background, Jackson Laboratory) used in this study were 6–10 week old and maintained under specific pathogen-free conditions.

### *C*. *albicans* and *T*. *cruzi* infections

*Trypanosoma cruzi* trypomastigotes (Tulahuen) were maintained by serial passages in WT mice. WT mice were i.p. infected with 5 × 10^5^ trypomastigotes from *T*. *cruzi* diluted in PBS. Mice were euthanized between days 14 and 16 post-infection. *Trypanosoma cruzi* parasites (Y-Br strain) were cultured in NIH3T3 mouse fibroblasts and were collected as described[60]. Mice 7–9 weeks of age were infected by intraperitoneal injection of 1 × 10^4^ trypomastigotes, diluted in a solution of 1% glucose in PBS[60].

Yeast cells of *C*. *albicans* were grown on Sabouraud glucose agar slopes at 28°C, and maintained by weekly subculture. B6 mice were i.p. injected with 3 × 10^7^ viable yeast diluted in PBS. Mice were sacrificed 5 days after the infection.

### Hydrodynamic cDNA injections

The hydrodynamic gene transfer procedure was described previously by our laboratory[7, 35, 36]. The designated amount of each DNA was dissolved in 1.6 mL of sterile 0.9% sodium chloride solution. Animals were injected in the tail vein with the cDNAs in less than 8 s and separated in two groups, control: 15 μg of ORF empty vector control cDNA and IL-12 + IL-18: 1 μg of IL-12 cDNA (pscIL-12, p40-p35 fusion gene) plus 10 μg of IL-18 cDNA (pDEF pro-IL-18). All the expression plasmids utilize the human elongation 1-α promoter to drive transcription.

### Flow cytometry, cell sorting, tetramers and FTY720 treatment

For multicolor staining, fluorochrome-conjugated Abs (BDPharmingen, Immuno tools, Ligatis, Miltenyi Biotec) were used in various combinations. Briefly, cells were stained for surface markers (CD4, CD8, CD11b, CD11c, CD44, NK1.1, CD45.1, CD45.2, NKG2D, CD122, CD124 (IL4R), CD49d for 30 min at 4°C and washed twice. To detect intracellular expression of cytokines or granzyme production, cells were cultured with PMA (50ng/ml) and Ionomycin (1μg/ml) for 4 h and 5 μg/ml Brefeldin A (Sigma) was added during the last 3 h. Cells were then stained for surface markers, washed, and fixed with Cytofix/Cytoperm buffer (BD Pharmingen) for 30 min at 4°C. Cells were washed with Perm Wash buffer (BD Pharmingen) and incubated with the anti-mouse IFNγ Ab or isotype-matched Ab for 30 min at 4°C. Following two washings, cells were analyzed in the flow cytometer. To detect intracellular IL-4 and intranuclear EOMES, Tbet or PLZF expression, cells were stained for surface markers, washed, and fixed with IC Fixation Buffer (eBioscience) for 90 minutes at 4°C. Cells were washed with Permeabilization Buffer (eBioscience) and incubated for 30 minutes with the same buffer. Cells were centrifuged and incubated with the Eomes PE anti-mouse Ab, Tbet PCP-Cy5.5 or PECy7 anti-mouse Ab, PLZF PECy7 anti-mouse Ab or isotype-matched Ab (BD-Pharmingen) for 45 min at 4°C and then analyzed by flow cytometry in a BD FACS CantoTM II cytometer or BD LSR Fortessa X-20 cytometer (BD Biosciences, San José, CA, USA). For cell sorting, cells were stained with monoclonal Abs and separated in a Becton Dickinson FACSAria II cytometer (BD Biosciences, San José, CA, USA) as SP4 CD44^+^ cells and NKT cells (See gate strategy in S3 Fig). Then, cells (10 x 10^5^ cells/100μl/well) were *in vitro* stimulated with PMA (50ng/ml) and Ionomycin (1μg/ml) for 5 h. Supernatant were harvested and IL-4 production was measured by ELISA following the manufactures´ instructions.

*T*. *cruzi*-specific CD8^+^ T cells were detected using H-2K(b) *T*. *cruzi* trans-sialidase amino acids 567–574 ANYKFTLV (TSKB20) APC-labeled Tetramer (NIH Tetramer Core Facility). OVA-specific CD8^+^ T cells were detected using H-2K(b) chicken ova amino acids 257–264 SIINFEKL APC-labeled Tetramer (NIH Tetramer Core Facility). NKT cells were detected using CD1d APC-labeled Tetramer (NIH Tetramer Core Facility).

For FTY720 *in vivo* treatment, control or *T*. *cruzi*-infected B6 mice (Tulahuen) were i.p. injected with 25μg of FTY720 (Sigma-Aldrich) resuspended in 200μl sterile 0.9% sodium chloride solution on days 8, 10 and 13 post-infection (pi) based on a previous report[37]. Mice were euthanized on day 14 pi.

### Immunofluorescence staining

To visualize *T*. *cruzi* parasites by immunofluorescence, thymi from WT mice were harvested 14–16 days post infection and a bulk suspension of thymocytes was resuspended in complete medium (RPMI 1640 supplemented with 10% heat-inactivated FBS, 100 U/ml penicillin G sodium, 100 μg/ml streptomycin sulfate, 2 mM L-glutamine, 1 mM sodium pyruvate, 1x essential amino acids, and 10 mM 2-ME). The bulk population of thymocytes were then placed into 24 well plates with glass slides inside and cultured for 72h at 37°C, 5% CO_2_. After the incubation period, adherent cells were enriched on the glass slides by washing the non-adherent cells with warm supplemented media. Slices were then stained with a serum from a chagasic patient along with a primary rat IgG antibody anti-CD11b[61]. Subsequently, the samples were incubated with a secondary anti-human IgG conjugated with FITC and anti-rat IgG conjugated to Alexa Fluor 546. Finally, the cells were stained with DAPI (300 ng/ml) to distinguish cell nuclei. The images were taken on a confocal microscope Olympus-1000 spectral Fluoview.

### Cytokine analysis

Culture supernatants were assayed for mIL-4 production by ELISA (BD-Pharmingen, La Jolla, CA) according to the manufacturers’ instructions.

### Proliferation assay

Thymi were mechanically disrupted, washed, and resuspended in supplemented medium (RPMI 1640 supplemented with 10% heat-inactivated FBS, 100 U/ml penicillin G sodium, 100 μg/ml streptomycin sulfate, 2 mM L-glutamine, 1 mM sodium pyruvate, 1 x essential amino acids, and 10 mM 2-ME). Cells were counted and stained with 4mM CFSE dye and then cultured at 1x10^6^ cells/ml at 37°C with medium alone or in the presence of IL-15 (150 ng/ml), IL-4 (20ng/ml), IL-12 (100 ng/ml) plus IL-18 (50 ng/ml), for 72 h, in triplicate in 96-well flat-bottom plates. Cells were stained for surface markers for 30 min at 4°C and washed twice and then gated for CSFE dilution analysis by flow cytometry in a BD FACS Canto TM II cytometer (BD Biosciences, San Jose, CA, USA).

### Evaluation of the degranulation marker CD107a

Thymi were mechanically disrupted, washed, and resuspended in supplemented medium (RPMI 1640 supplemented with 10% heat-inactivated FBS, 100 U/ml penicillin G sodium, 100 μg/ml streptomycin sulfate, 2 mM L-glutamine, 1 mM sodium pyruvate, 1 x essential amino acids, and 10 mM 2-ME). Cells were counted and cultured at 2x10^6^ cells/ml at 37°C with medium in the presence of PMA (50 ng/ml), ionomycin (1μg/ml), GolgiStop (BD Biosciences) and anti-CD107a antibody (0,002 μg/μl) for 5 hours in 96-well flat-bottom plates. Cells were stained for surface markers for 30 min at 4°C and washed twice and then analyzed by flow cytometry in a BD FACS CantoTM II cytometer (BD Biosciences, San Jose, CA, USA).

### Intrathymic injections and IL-15 *in vivo* neutralization

Intrathymic injections were performed in 8-wk-old C57BL6 CD45.2^+^ or CD45.1^+^ WT mice. Briefly, mice were anesthetized by i.p. injection of ketamine (0,05mg/g) and xylazine (0,01mg/g) (both from Richmond Vet Pharma) in saline. An incision was opened to expose the thymus, and 10 μl of eFluor 670 (eF670) dye (0,5mM, BD Biosciences) or 10 × 10^6^ thymocytes from OT-I mice (97–99% Vβ5^+^) resuspended in 20 μl PBS, were injected into CD45.1^+^ or CD45.2^+^ B6 recipient mice. The wound was closed with instant adhesive, and the mice were placed in a warm environment until they recovered. Mice were analyzed 6 days after eF670 injection or 48h after cell injections.

For IL-15 *in vivo* neutralization, IL-4 KO *T*. *cruzi*-infected (Tulahuen) mice were i.p. injected with 25μg of an anti-IL15 (eBioscience) resuspended in 200μl sterile 0.9% sodium chloride solution on days 10 and 12 post-infection (pi). Mice were euthanized on day 14 pi.

### Adoptive transfer (AT) experiments

Thymi from *T*. *cruzi-*infected (Tulahuen) WT mice or IL-12 + IL-18-injected mice were obtained and cell suspensions were prepared. Approximately 5–6 × 10^6^ total thymocytes were resuspended in 0.2 mL of sterile 0.9% sodium chloride solution and injected i.v into the B6 recipient’s retrorbital sinus. For AT of OT-I (97–99% OVA-tetramer^+^ Vβ5^+^) thymocytes from *T*. *cruzi-*infected mice, SP8 cells were sorted and approximately 5–6 x 10^6^ cells were AT to recipient B6 mice as described above. Three hours post-adoptive transfer, recipient mice were infected i.p. with 5000 trypomastigotes from *T*. *cruzi* (Tulahuen) diluted in 200 μl PBS and monitored for the number of parasites (per ml of blood) in peripheral blood at 10, 13 and 16 days post infection (dpi) with survival measured during 50 days. The control group was B6 mice infected i.p. with 5000 parasites (Tulahuen) without the cell transfer.

### Co-cultures experiments

Thymi from CD45.1^+^ control WT mice, CD45.2^+^ *T*. *cruzi*-infected (Tulahuen) WT mice and CD45.2^+^ *T*. *cruzi-*infected (Tulahuen) IL-4KO mice were obtained and thymocytes isolated and resuspended in PBS + 5% FBS. Cells were counted and then stimulated with PMA (50 ng/ml) and Ionomycin (1 μg/ml) for 2 hours in 24-well culture plates by placing 1x 10^6^ cells in each well in 1 ml of complete RPMI (10% 1/100 Glutamine, 1/1000 Gentamycin). Stimulated cells were then washed twice and in the corresponding reservoirs, an anti-IL15 mAb (100 μg/ml, eBioscience) was added to block IL-15. Secondly, 1 x 10^6^ cells from the bulk population of thymocytes or, bulk DP (CD4^+^CD8^+^ cells), DP (CD4^+^CD8^+^ CD69^neg^ cells), DP (CD4^+^CD8^+^ CD69^pos^ cells) or SP8 CD44^low^ sorted cells from CD45.2^+^ OT-I control mice were extracted and co-cultured with the previously mentioned cells in a 1:1 ratio. The co-cultures were maintained at 37°C under a constant atmosphere of 5% CO_2_. After 48h, cells were removed from the plate and analyzed by flow cytometry.

### mRNA extraction and analysis

Total RNA was isolated using a single-step phenol/chloroform extraction procedure (TRIzol; Invitrogen Life Technologies). Real-time (RT) PCR was performed with 100 ng of total RNA for each sample (Super Script III one step RT-PCR with platinum Taq, Invitrogen), utilizing the following program: 15 min reverse transcription at 45°C, 40 cycles of denaturing at 94°C (15 s), annealing at 55°C (30 s), and extension at 68°C (60 s), with a final extension for 5 min at 68°C. Primers used were: IL-15Ra S: 5'-CCCACAGTTCCAAAATGACGA-3'; AS: 5'-GCTGCCTTGATTTGATGTACCAG-3'. IL-15 S: 5'-ACATCCATCTCGTGCTACTTGT-3'; AS: 5'-GCCTCTGTTTTAGGGAGACCT-3'. Briefly, RNAs were treated with DNase I prior to reverse transcription. Reverse transcription was performed on 1 μg of RNA using random hexamers as primers. Semiquantitative real time PCR was performed on cDNAs using TaqMan R expression assays (Life Technologies) specific for each target gene. All reactions were run on a 96-well, 7300 Real Time PCR System (Life Technologies). Expression of all target genes was normalized using HPRT or GAPDH as the control housekeeping gene. For IL-15 expression, 5 μg of total cytoplasmic RNA was analyzed using the RiboQuan kit mCK.1 (BD Pharmingen) and [33P]UTP-labeled riboprobes by the RNase protection assay (RPA).

### Statistical analysis

Data were compared in all cases between each treated-mice group with its own control group. Results are expressed as means ± SEM. Data were analyzed using one-way analysis of variance (ANOVA) with a Bonferroni post-test to compare different columns (p < 0.05). In all cases, the assumptions of ANOVA (homogeneity of variance and normal distribution) were attained. When indicated, significant differences were performed using Student’s t test for paired or grouped samples. For survival analysis the statistic test applied was Gehan-Brelow-Wilcoxon test. In all statistical analyses, p < 0.05 was considered to represent a significant difference between groups.

## Supporting information

S1 Fig OT-I SP8 thymocytes adopt an innate phenotype after *T*. *cruzi* infection. Thymocytes from WT and OT-I control or *T*. *cruzi*-infected (Tulahuen) mice were obtained 14 days after infection. (A) The expression of CD122, CD49d, Eomes and Tbet was evaluated by Flow cytometry in the SP8 CD44hi subset from OT-I control or *T. cruzi*-infected mice (B) The percentage and absolute number of SP8 CD44lo and SP8 CD44hi cells was calculated. CD44hi Control vs CD44hi *T. cruzi p<0.01 and p<0.001*. Eomes or Tbet were measured by intranuclear staining using Flow cytometry analysis and were expressed as the difference of the mean fluorescence intensity (MFI) of Eomes or Tbet vs the MFI of the correspondent isotype control (IC) in the SP8 CD44^hi^ subset. Data is expressed as a representative histogram and bars (mean ± SEM) from three repetitions of the same experiment with 3–5 animals per group. The statistical test applied was a One-way ANOVA. Index data are shown as the mean ± SEM. Control vs T-cruzi **p<0.01 and ***p<0.*001*. TF = transcription factor.
(TIF)

S2 Fig SP8 thymocytes adopt an innate phenotype in different infectious/inflammatory models. (A) Thymocytes from WT *C*. *albicans*-infected mice were obtained 5 days after infection. Eomes expression was measured by intranuclear staining using Flow cytometry analysis in the SP4 and SP8 cells, CD44^hi^ or CD44^lo^ obtained. The index value was obtained by dividing the mean fluorescence intensity (MFI) of the EOMES vs the MFI of the isotype control. Data is the result of three repetitions of the same experiment with 3–5 animals per group. The statistical test applied was One-way ANOVA. SP8 CD44^hi^ vs SP8 CD44l^o^ *p<0.*05*.(B) B6 mice were infected with *T*. *cruzi* (Y strain) and thymi were harvested on day 14 post-infection. Evaluation of innate markers CD122, CD49d and Eomes was analyzed by flow cytometry in the increased SP8 CD44^hi^ population. Data represents one experiment with 5 mice per group. The statistical test applied was a Student’s unpaired *t* test. (C) B6 mice were hydrodynamically injected with control or IL-12+IL-18 cDNAs. Thymocytes were obtained 7 days post-injections. The expression of CD122 and CD49d was evaluated by Flow cytometry in the SP8 CD44^hi^ cell subset. Eomes and Tbet were measured by intranuclear staining using Flow cytometry analysis and were expressed as the difference of the mean fluorescence intensity (MFI) of Eomes or Tbet vs the MFI of the correspondent isotype control (IC) in the SP8 CD44^hi^thymocytes. Data is expressed as a representative histogram and bars (mean ± SEM) from three repetitions of the same experiment with 3–5 animals per group. The statistical test applied was One-way ANOVA. Control vs T-cruzi*p<0.*05* and **p<0.*01*. TF = transcription factor. The statistical test applied was One-way ANOVA. Index data are shown as the mean ± SEM. Control versus 12+18, *p<0.*05*.
(TIF)

S3 Fig Gate strategy used to perform IL-4 intracellular analysis and cell sort of thymic NKT and SP4 CD44^hi^ cells. (TIF)

S4 Fig High IFNγ production by innate CD8^+^ thymocytes correlates with Eomes expression. A bulk population of thymocytes from control or *T*. *cruzi-*infected OT-I mice were obtained at day 14 post-infection and cultured for 5h a 37°C in the presence of PMA/ionomycin and in the last 3 hours in the presence of monensin. After that, thymocytes were obtained and Eomes expression and the IFNγ production were analyzed by Flow cytometry in SP8 CD44^hi^ and SP8 CD44^lo^ OVA-tetramer^+^ thymocytes. Dot plots are representative of 2 independent experiments with 3–5 mice/group. The statistical test applied was a Student’s unpaired *t* test.
(TIF)

S5 Fig Innate CD8^+^ cells appearance in the thymus is a SP8 lineage decision. WT mice were infected with *T*. *cruzi* (Tulahuen) or left uninfected (control). At day 7 post-infection, (A) some of the mice were euthanized, thymocytes were obtained and CD44, CD122, CD49d, Eomes and Tbet expression were analyzed by Flow cytometry only in the SP8 subset or (B) the rest of the mice were anaesthetized and intrathymically (i.t.) injected with 8 μl (0,5mM) of eFluor 670 dye (eF 670). Seven days later (day 14 post-infection) the thymi were harvested. Dot plot show the representative gate strategy of one mouse per group. The percentage of CD44^hi^ cells was analyzed by Flow cytometry in the eF 670^+^ SP8 thymocytes. Data is expressed as mean ± SEM of three independent experiments with 3–5 mice per group. The statistical test applied was a Student’s unpaired *t* test, Control vs *T*. *cruzi*-infected *p<0.*05*.
(TIF)

S6 Fig Thymocytes from *T*. *cruzi*-infected mice induce *in vitro* large numbers of SP8 cells from DP cells. A bulk population of CD45.2^+^ WT control or WT *T*. *cruzi-*infected (Tulahuen) mice were obtained at day 14 post-infection and cultured for 2h at 37°C in the presence of PMA/ionomycin. Cells were washed twice and co-cultured at a 1:1 ratio with either DP cells sorted from a CD45.1^+^ WT or DP CD69^+^ or DP CD69^-^ cells sorted from a CD45.2^+^ OT-I control mice. (A) Gate strategy to separate DP CD69^+^ or DP CD69^-^ cells from OTI mice. After 48h, cells were obtained and (B) representative density plots are shown from two independent experiments with 4–6 mice/group. The statistical test applied was One-way ANOVA. Control vs *T*. *cruzi*-infected **p<0.*01*.
(TIF)

S7 Fig The blockage of IL-4 and IL-15 inhibits the induction of the innate phenotype in SP8 cell generated in vitro from DP thymocytes. A bulk population of thymocytes from WT control, WT *T*. *cruzi-*infected (Tulahuen) or IL-4KO *T*. *cruzi-*infected (Tulahuen) mice were obtained at day 14 post-infection and cultured for 2h at 37°C in the presence of PMA/ionomycin. Cells were washed twice and co-cultured with sorted DP cells from OT-I control mice at a 1:1 ratio in the presence or absence of a neutralizing anti-IL-15 Ab. After 48h, thymocytes were obtained and CD44, CD122, CD49d, Eomes and Tbet expression were analyzed by Flow cytometry only in the SP8 OT-I thymocytes generated “*in vitro*” from DP OT-I T cells (Vβ5^+^ OVA-tetramer^+^). Eomes or Tbet were measured by intranuclear staining using Flow cytometry analysis. Histograms are representative of two independent experiments with 3–6 mice/group. The statistical test applied was a One-way ANOVA. *T*. *cruzi* vs the rest of the groups, *p<0.*05*. Tc = *T*. *cruzi*; Tc+α15 = *T*. *cruzi* + anti-IL-15 neutralizing Ab; Tc4KO = IL-4 KO *T*. *cruzi*; Tc4KO+α15 = IL-4 KO *T*. *cruzi* + anti-IL-15 neutralizing Ab.
(TIF)

S8 Fig *In vitro* blocking of IL-4 and IL-15 are unable to revert the induction of the innate phenotype in OT-I sorted SP8 thymocytes. A bulk population of WT control, WT *T*. *cruzi-*infected (Tulahuen) or IL-4KO *T*. *cruzi-*infected (Tulahuen) mice were obtained at day 14 post-infection and cultured for 2h at 37°C in the presence of PMA/ionomycin. Cells were washed twice and co-cultured with sorted SP8 cells from OT-I control mice at a 1:1 ratio in the presence or absence of a neutralizing anti-IL-15 Ab. After 48h, thymocytes were obtained and CD44, CD122, CD49d, expression was analyzed by Flow cytometry only in SP8 OVA-specific OT-I thymocytes (Vβ5^+^ OVA-tetramer^+^). Eomes or Tbet were measured by intranuclear staining using Flow cytometry analysis. Histograms are representative of two independent experiments with 3–6 mice/group. The statistical test applied was One-way ANOVA. IC = Isotype control; Tc = *T*. *cruzi*; Tc+α15 = *T*. *cruzi* + anti-IL-15 neutralizing Ab; Tc4KO = IL-4 KO *T*. *cruzi*; Tc4KO+α15 = IL-4 KO *T*. *cruzi* + anti-IL-15 neutralizing Ab.
(TIF)
