# Supplementary figures and images for: Thymic expression of IL-4 and IL-15 after systemic inflammatory or infectious Th1 disease processes induce the acquisition of "innate" characteristics during CD8+ T cell development
Source: PLoS Pathog. 2019 Jan 4;15(1):e1007456. doi: 10.1371/journal.ppat.1007456 (PMC6319713; doi:10.1371/journal.ppat.1007456)

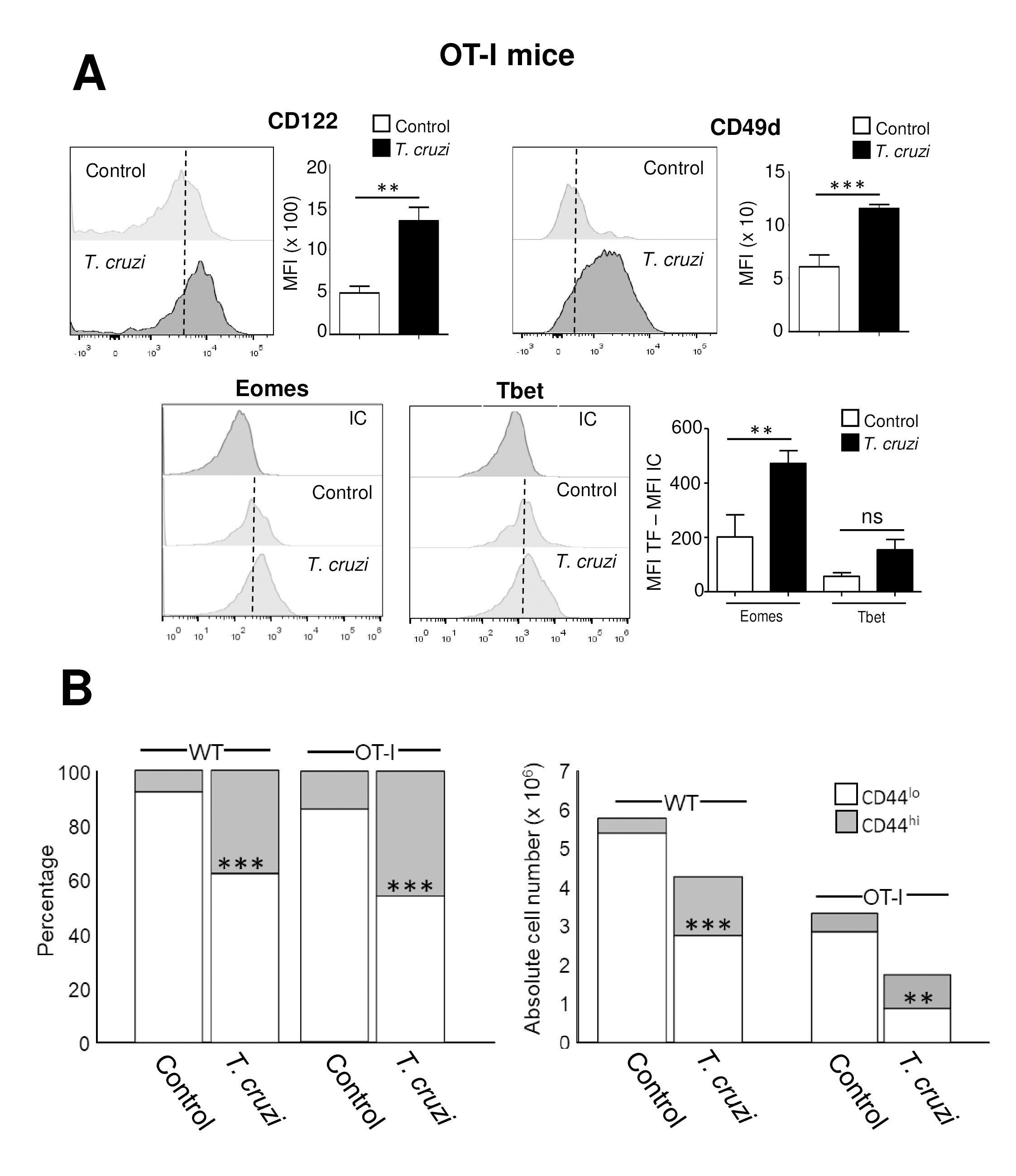

Supplement: S1 Fig — Thymocytes from WT and OT-I control or T. cruzi-infected (Tulahuen) mice were obtained 14 days after infection. (A) The expression of CD122, CD49d, Eomes and Tbet was evaluated by Flow cytometry in the SP8 CD44hi subset from OT-I control or T. cruzi-infected mice (B) The percentage and absolute number of SP8 CD44lo and SP8 CD44hi cells was calculated. CD44hi Control vs CD44hi T. cruzi p<0.01 and p<0.001. Eomes or Tbet were measured by intranuclear staining using Flow cytometry analysis and were expressed as the difference of the mean fluorescence intensity (MFI) of Eomes or Tbet vs the MFI of the correspondent isotype control (IC) in the SP8 CD44hi subset. Data is expressed as a representative histogram and bars (mean ± SEM) from three repetitions of the same experiment with 3–5 animals per group. The statistical test applied was a One-way ANOVA. Index data are shown as the mean ± SEM. Control vs T-cruzi **p<0.01 and ***p<0.001. TF = transcription factor. (TIF) [file ppat.1007456.s001.tif]

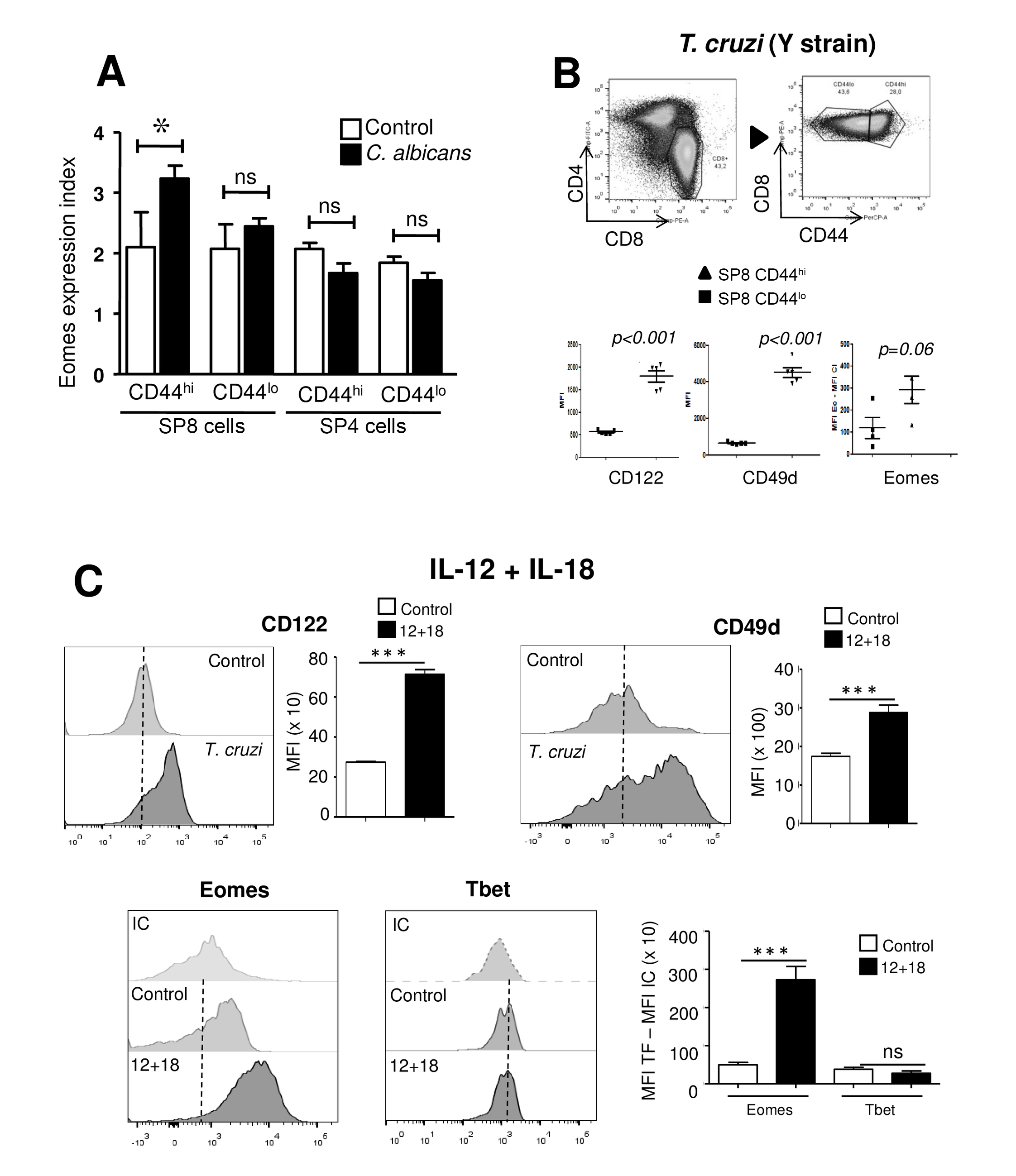

Supplement: S2 Fig — (A) Thymocytes from WT C. albicans-infected mice were obtained 5 days after infection. Eomes expression was measured by intranuclear staining using Flow cytometry analysis in the SP4 and SP8 cells, CD44hi or CD44lo obtained. The index value was obtained by dividing the mean fluorescence intensity (MFI) of the EOMES vs the MFI of the isotype control. Data is the result of three repetitions of the same experiment with 3–5 animals per group. The statistical test applied was One-way ANOVA. SP8 CD44hi vs SP8 CD44lo *p<0.05.(B) B6 mice were infected with T. cruzi (Y strain) and thymi were harvested on day 14 post-infection. Evaluation of innate markers CD122, CD49d and Eomes was analyzed by flow cytometry in the increased SP8 CD44hi population. Data represents one experiment with 5 mice per group. The statistical test applied was a Student’s unpaired t test. (C) B6 mice were hydrodynamically injected with control or IL-12+IL-18 cDNAs. Thymocytes were obtained 7 days post-injections. The expression of CD122 and CD49d was evaluated by Flow cytometry in the SP8 CD44hi cell subset. Eomes and Tbet were measured by intranuclear staining using Flow cytometry analysis and were expressed as the difference of the mean fluorescence intensity (MFI) of Eomes or Tbet vs the MFI of the correspondent isotype control (IC) in the SP8 CD44hithymocytes. Data is expressed as a representative histogram and bars (mean ± SEM) from three repetitions of the same experiment with 3–5 animals per group. The statistical test applied was One-way ANOVA. Control vs T-cruzi*p<0.05 and **p<0.01. TF = transcription factor. The statistical test applied was One-way ANOVA. Index data are shown as the mean ± SEM. Control versus 12+18, *p<0.05. (TIF) [file ppat.1007456.s002.tif]

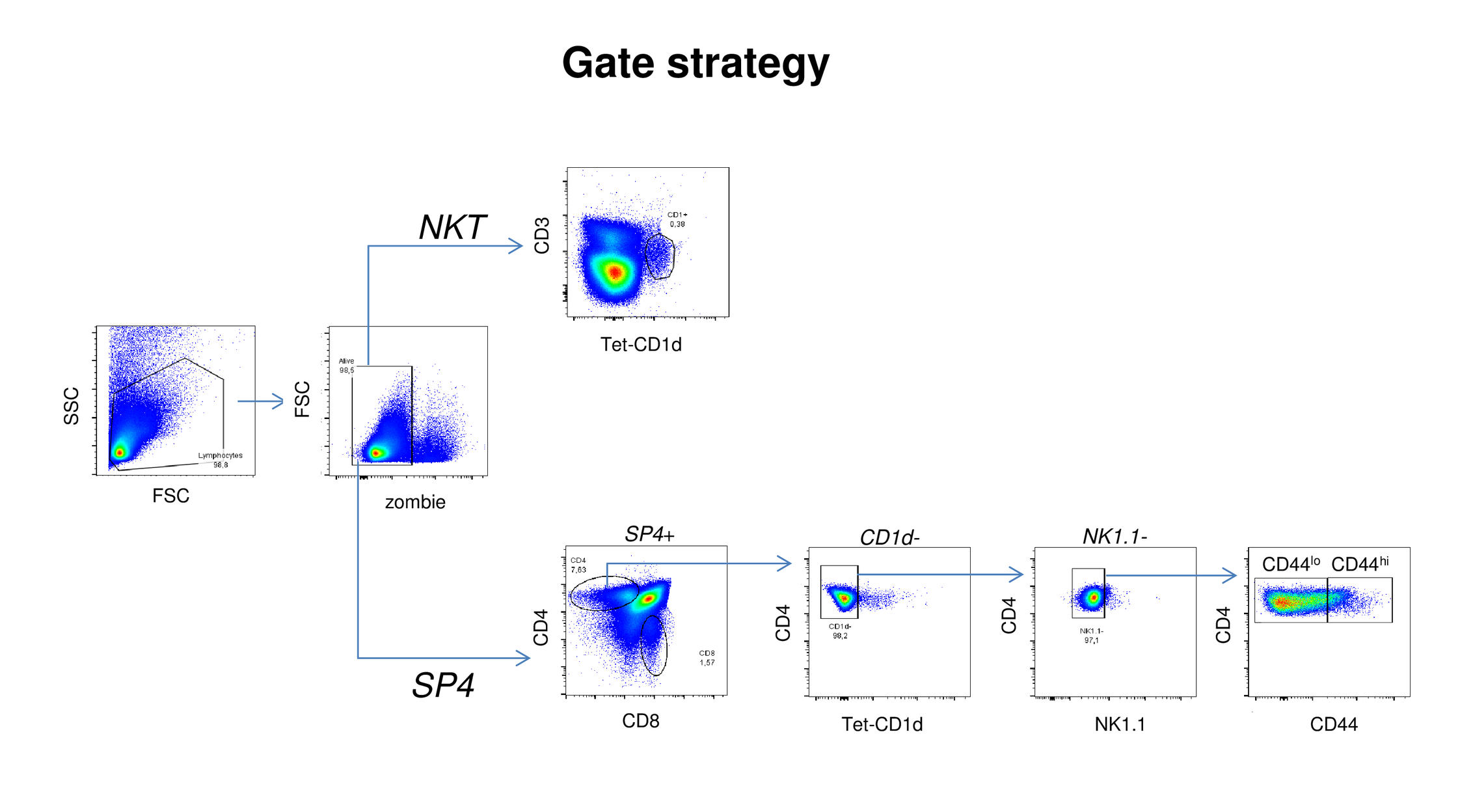

Supplement: S3 Fig — (TIF) [file ppat.1007456.s003.tif]

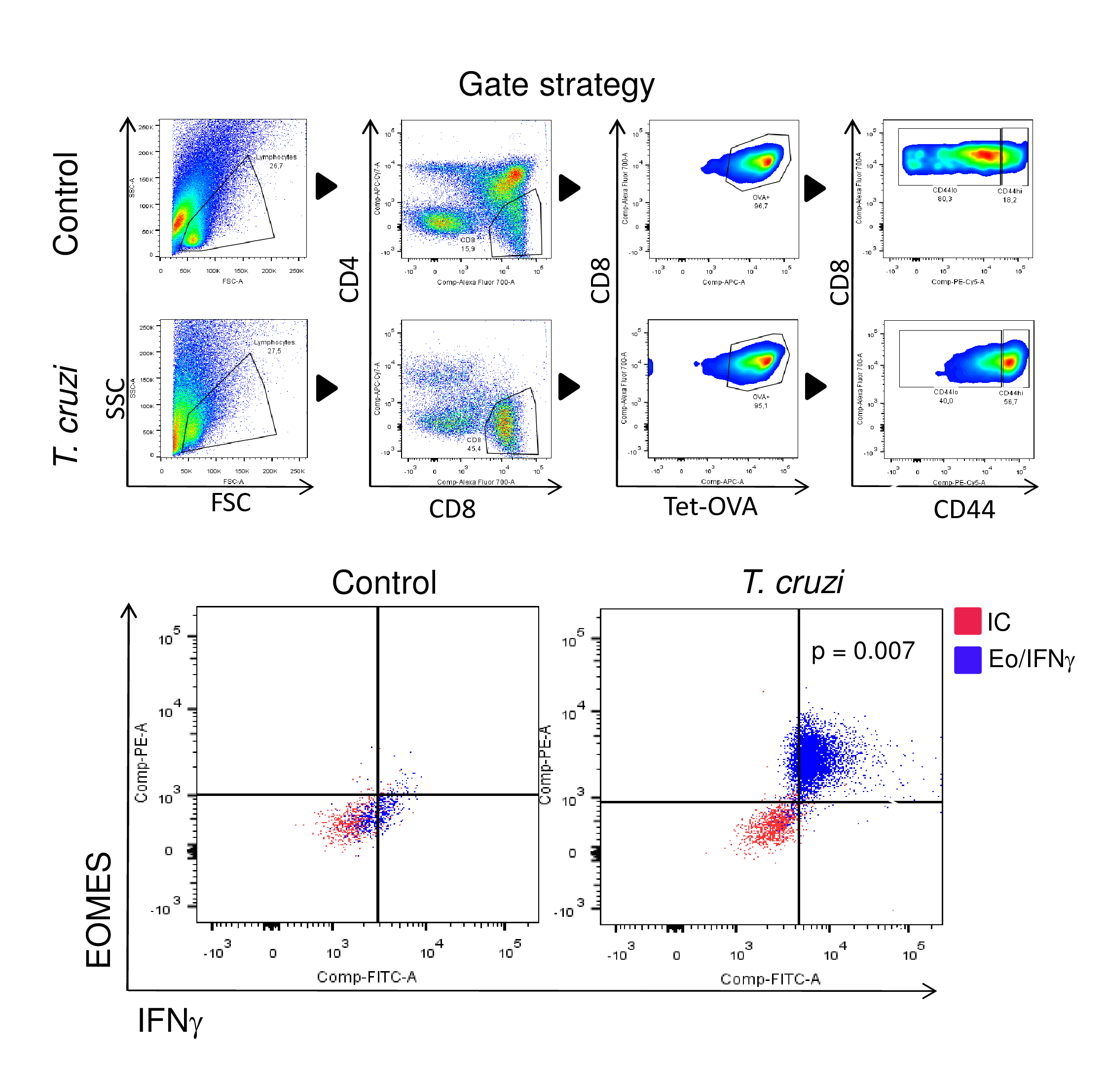

Supplement: S4 Fig — A bulk population of thymocytes from control or T. cruzi-infected OT-I mice were obtained at day 14 post-infection and cultured for 5h a 37°C in the presence of PMA/ionomycin and in the last 3 hours in the presence of monensin. After that, thymocytes were obtained and Eomes expression and the IFNγ production were analyzed by Flow cytometry in SP8 CD44hi and SP8 CD44lo OVA-tetramer+ thymocytes. Dot plots are representative of 2 independent experiments with 3–5 mice/group. The statistical test applied was a Student’s unpaired t test. (TIF) [file ppat.1007456.s004.tif]

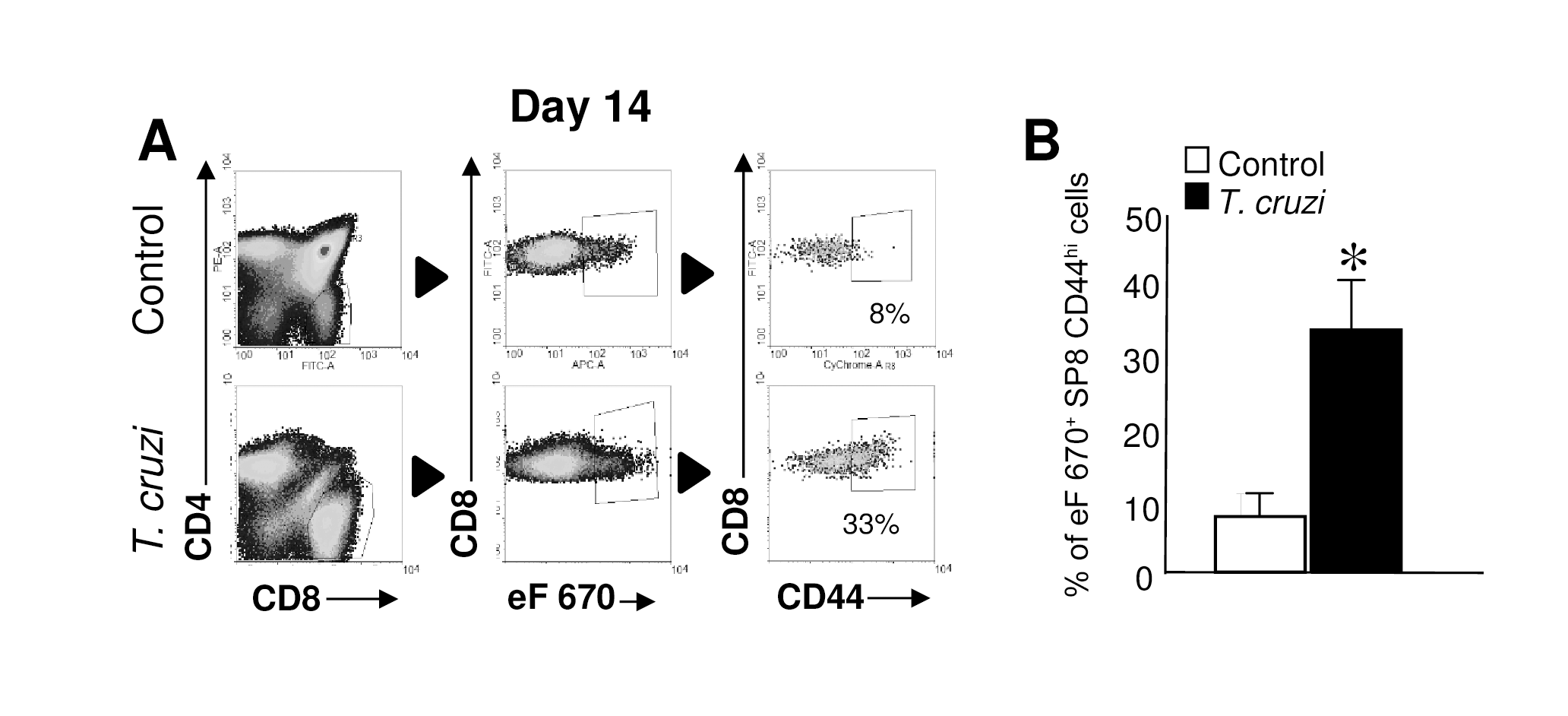

Supplement: S5 Fig — WT mice were infected with T. cruzi (Tulahuen) or left uninfected (control). At day 7 post-infection, (A) some of the mice were euthanized, thymocytes were obtained and CD44, CD122, CD49d, Eomes and Tbet expression were analyzed by Flow cytometry only in the SP8 subset or (B) the rest of the mice were anaesthetized and intrathymically (i.t.) injected with 8 μl (0,5mM) of eFluor 670 dye (eF 670). Seven days later (day 14 post-infection) the thymi were harvested. Dot plot show the representative gate strategy of one mouse per group. The percentage of CD44hi cells was analyzed by Flow cytometry in the eF 670+ SP8 thymocytes. Data is expressed as mean ± SEM of three independent experiments with 3–5 mice per group. The statistical test applied was a Student’s unpaired t test, Control vs T. cruzi-infected *p<0.05. (TIF) [file ppat.1007456.s005.tif]

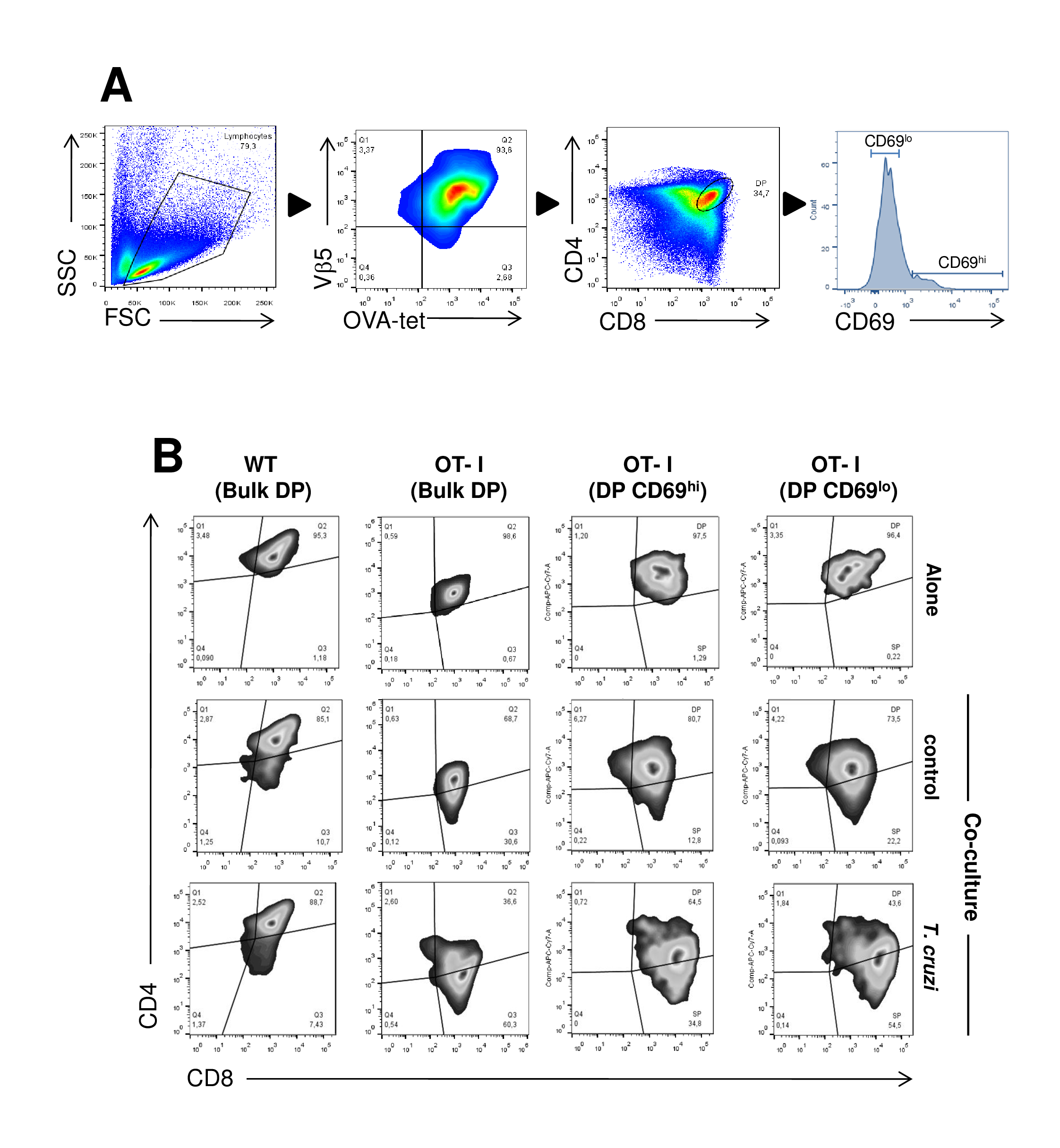

Supplement: S6 Fig — A bulk population of CD45.2+ WT control or WT T. cruzi-infected (Tulahuen) mice were obtained at day 14 post-infection and cultured for 2h at 37°C in the presence of PMA/ionomycin. Cells were washed twice and co-cultured at a 1:1 ratio with either DP cells sorted from a CD45.1+ WT or DP CD69+ or DP CD69- cells sorted from a CD45.2+ OT-I control mice. (A) Gate strategy to separate DP CD69+ or DP CD69- cells from OTI mice. After 48h, cells were obtained and (B) representative density plots are shown from two independent experiments with 4–6 mice/group. The statistical test applied was One-way ANOVA. Control vs T. cruzi-infected **p<0.01. (TIF) [file ppat.1007456.s006.tif]

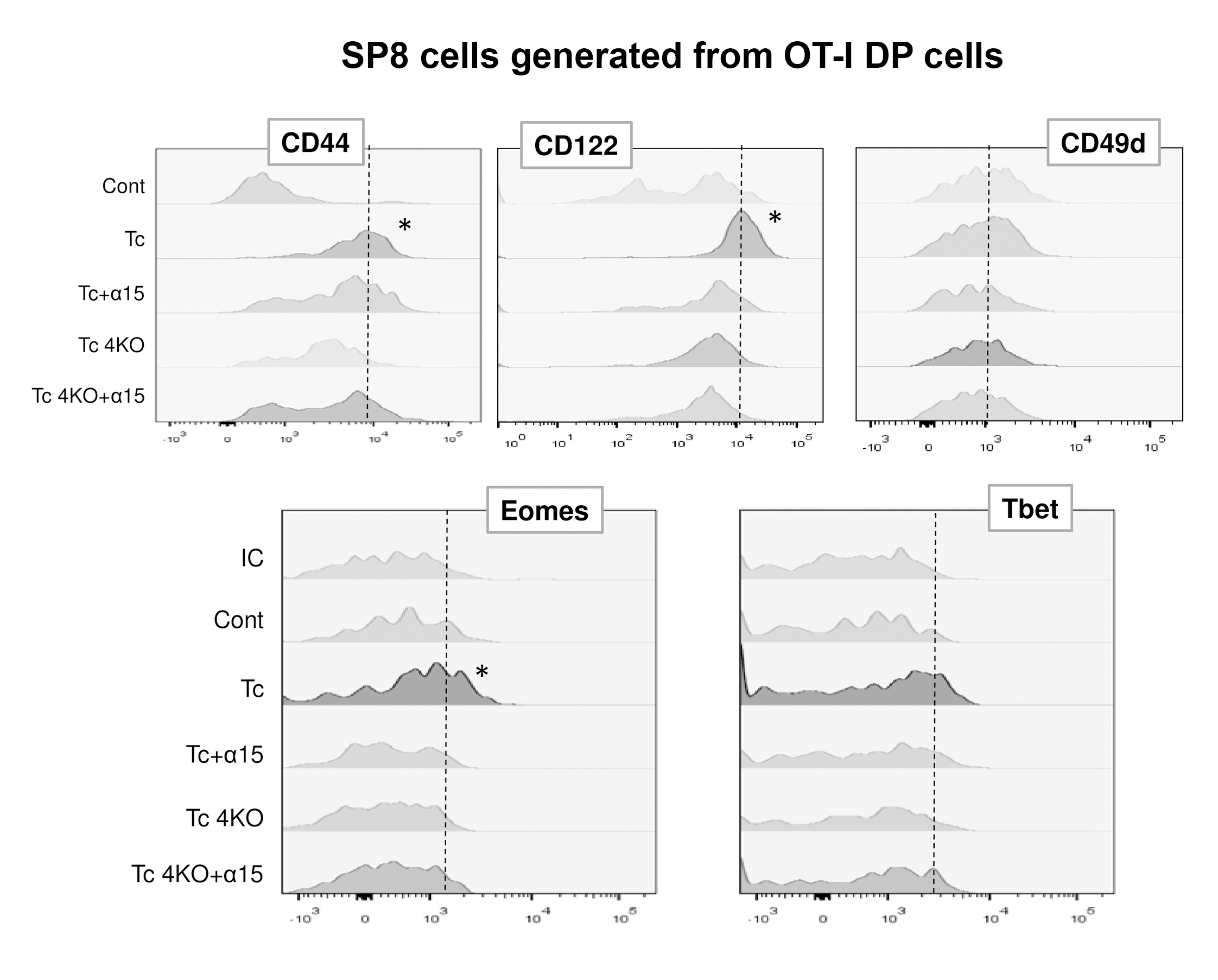

Supplement: S7 Fig — A bulk population of thymocytes from WT control, WT T. cruzi-infected (Tulahuen) or IL-4KO T. cruzi-infected (Tulahuen) mice were obtained at day 14 post-infection and cultured for 2h at 37°C in the presence of PMA/ionomycin. Cells were washed twice and co-cultured with sorted DP cells from OT-I control mice at a 1:1 ratio in the presence or absence of a neutralizing anti-IL-15 Ab. After 48h, thymocytes were obtained and CD44, CD122, CD49d, Eomes and Tbet expression were analyzed by Flow cytometry only in the SP8 OT-I thymocytes generated “in vitro” from DP OT-I T cells (Vβ5+ OVA-tetramer+). Eomes or Tbet were measured by intranuclear staining using Flow cytometry analysis. Histograms are representative of two independent experiments with 3–6 mice/group. The statistical test applied was a One-way ANOVA. T. cruzi vs the rest of the groups, *p<0.05. Tc = T. cruzi; Tc+α15 = T. cruzi + anti-IL-15 neutralizing Ab; Tc4KO = IL-4 KO T. cruzi; Tc4KO+α15 = IL-4 KO T. cruzi + anti-IL-15 neutralizing Ab. (TIF) [file ppat.1007456.s007.tif]

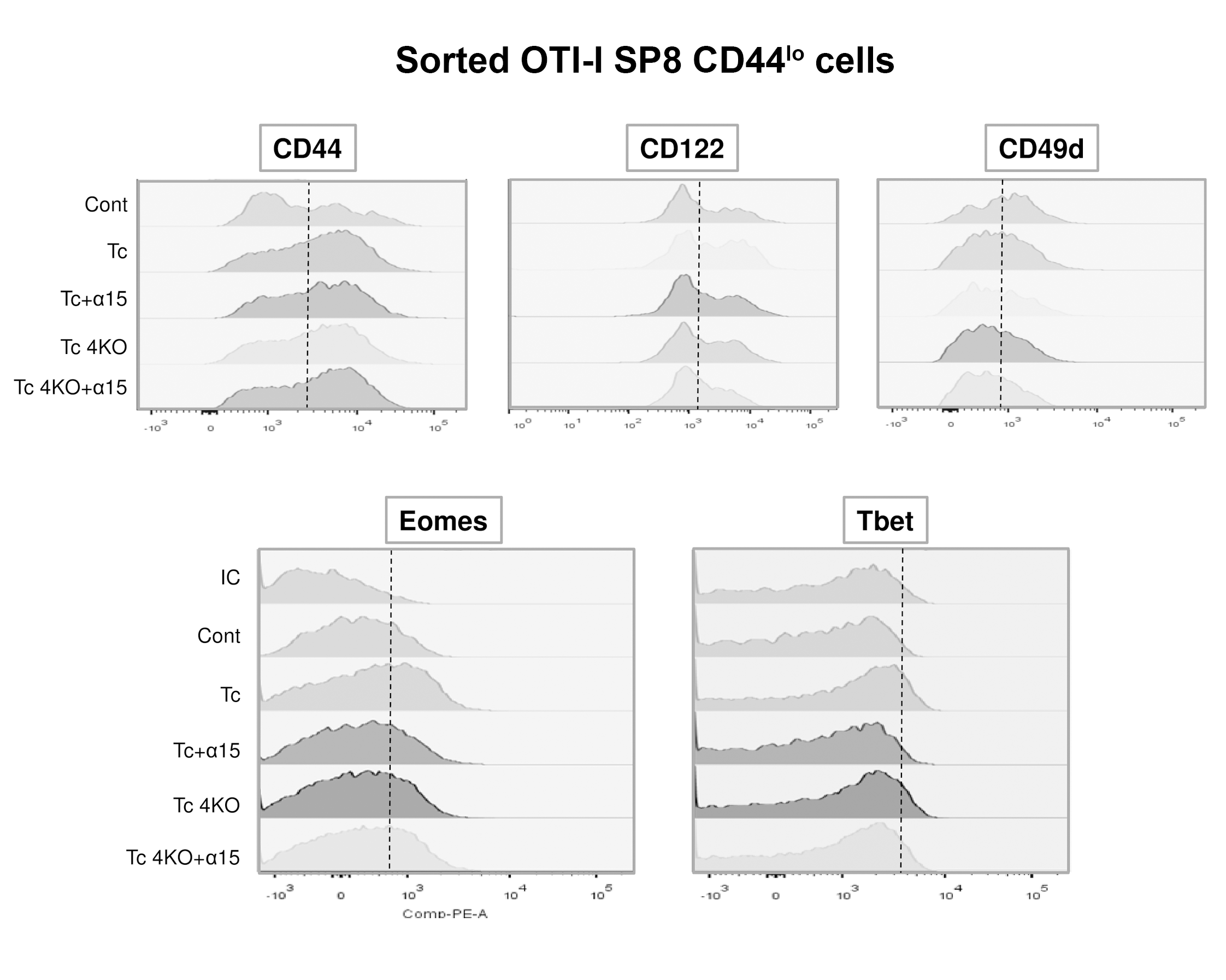

Supplement: S8 Fig — A bulk population of WT control, WT T. cruzi-infected (Tulahuen) or IL-4KO T. cruzi-infected (Tulahuen) mice were obtained at day 14 post-infection and cultured for 2h at 37°C in the presence of PMA/ionomycin. Cells were washed twice and co-cultured with sorted SP8 cells from OT-I control mice at a 1:1 ratio in the presence or absence of a neutralizing anti-IL-15 Ab. After 48h, thymocytes were obtained and CD44, CD122, CD49d, expression was analyzed by Flow cytometry only in SP8 OVA-specific OT-I thymocytes (Vβ5+ OVA-tetramer+). Eomes or Tbet were measured by intranuclear staining using Flow cytometry analysis. Histograms are representative of two independent experiments with 3–6 mice/group. The statistical test applied was One-way ANOVA. IC = Isotype control; Tc = T. cruzi; Tc+α15 = T. cruzi + anti-IL-15 neutralizing Ab; Tc4KO = IL-4 KO T. cruzi; Tc4KO+α15 = IL-4 KO T. cruzi + anti-IL-15 neutralizing Ab. (TIF) [file ppat.1007456.s008.tif]
